# Advances in CRISPR Base Editing: From Molecular Evolution to Therapeutic Applications in Genomic Medicine

**DOI:** 10.1111/jcmm.71159

**Published:** 2026-04-25

**Authors:** Melike Aliciaslan, Ezgi Erbasan, Fulya Erendor, Salih Sanlioglu

**Affiliations:** ^1^ Department of Gene and Cell Therapy, Faculty of Medicine Akdeniz University Antalya Turkey

**Keywords:** adenine base editor, base editing, cell therapy, CRISPR‐Cas9, cytosine base editor, gene therapy, genome engineering, medical genetics

## Abstract

CRISPR‐Cas9 systems revolutionized gene editing, but inherent drawbacks, namely DNA double‐strand breaks (DSBs) and the difficulty of achieving precise repairs (due to low HDR efficiency), led researchers to invent new, more accurate gene editing tools. Base editing represents a significant leap forward, enabling targeted single‐nucleotide conversions directly on the DNA without DSBs or donor templates. The core technology involves fusing catalytically dead or nickase Cas proteins to DNA deaminase enzymes. Cytosine base editors (CBEs) convert C•G to T•A pairs, while adenine base editors (ABEs) change A•T to G•C. These editors exploit the deaminase function within the R‐loop structure formed by Cas binding and co‐opt endogenous DNA repair mechanisms for precision. While offering improved efficiency and editing precision, base editing faces persistent challenges, such as off‐target effects, bystander edits, delivery and ethical concerns. Continuous engineering efforts have refined these tools, enhancing accuracy, expanding targetability and reducing unwanted edits. The base editing arsenal has also broadened to include C‐to‐G base editors (CGBEs), dual A&C editors and versions targeting organelles. Successful preclinical studies demonstrating the correction of mutations responsible for the disease have paved the way for clinical trials, which are now testing therapies for conditions like sickle cell disease, β‐thalassaemia and hypercholesterolemia using various delivery systems. This review explores CRISPR base editing's origins, mechanisms of action, potential therapies and current restrictions, pointing to its broadening impact on medical genetics.

## 1 Introduction

### 1.1 The Dawn of Precision Genome Editing

**FIGURE 1:**
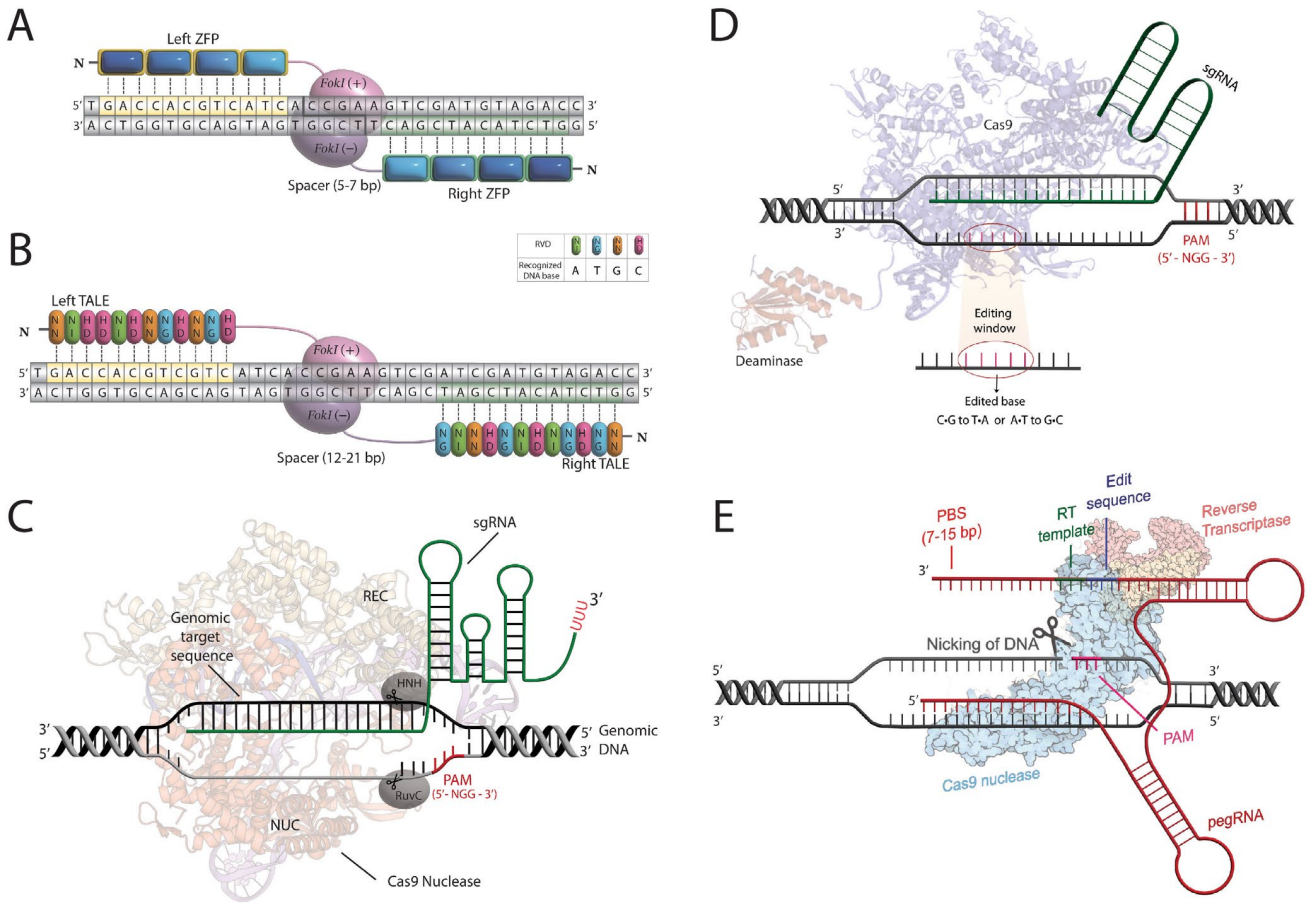
Comparison of Major Genome Editing Technologies. The figure illustrates five major classes of engineered nucleases and editing systems used for targeted modification of DNA sequences. (A) Zinc Finger Nucleases (ZNFs): Early engineered nucleases composed of modular zinc finger domains linked to a FokI nuclease domain. Each zinc finger domain recognizes approximately 3 base pairs of DNA. Pairs of ZNFs are designed to bind opposite strands of the target DNA site, allowing the FokI domains to dimerize and introduce a targeted double‐strand break (DSB). Cellular repair of the DSB via non‐homologous end joining (NHEJ) or homology‐directed repair (HDR) can result in gene disruption or correction, respectively. (B) Transcription Activator‐Like Effector Nucleases (TALENs): TALENs consist of DNA‐binding domains fused to a FokI nuclease. The DNA‐binding domains are derived from Transcription Activator‐Like Effectors (TALEs), where each module recognizes a single DNA base, offering potentially higher specificity and easier design compared to ZNFs. Like ZNFs, paired TALENs bind opposite strands to induce a targeted DSB, which is resolved by cellular repair pathways (NHEJ or HDR). (C) CRISPR‐Cas9 System: A revolutionary RNA‐guided nuclease system [4]. The Cas9 nuclease is directed to a specific DNA target site by a synthetic guide RNA (gRNA) which contains a sequence complementary to the target DNA. Binding specificity also requires a short Protospacer Adjacent Motif (PAM) sequence immediately downstream of the target. Upon binding, Cas9 introduces a blunt DSB, typically 3 base pairs upstream of the PAM. Subsequent cellular repair (NHEJ or HDR) leads to gene editing outcomes. (D) Base Editors (BEs): Derived from the CRISPR system, Base Editors enable precise single‐base changes without inducing DSBs [5]. They consist of a catalytically impaired Cas protein (either a nickase, nCas9, which cuts only one strand, or a deactivated Cas9, dCas9, which doesn't cut at all) fused to a DNA deaminase enzyme (e.g., cytidine deaminase for C•G to T•A edits or adenine deaminase for A•T to G•C edits). Guided by an sgRNA, the editor binds to the target DNA, and the deaminase directly converts a target base within a specific editing window. A nick on the non‐edited strand often enhances the efficiency of incorporating the edit during DNA repair. (E) Prime Editors (PEs): A versatile ‘search‐and‐replace’ genome editing technology that mediates targeted insertions, deletions, and all 12 possible base‐to‐base conversions without requiring DSBs or donor DNA templates [6]. Prime Editors consist of a Cas9 nickase fused to a reverse transcriptase (RT). They are programmed with a prime editing guide RNA (pegRNA) that both specifies the target site and contains an RT template encoding the desired edit. The PE nicks one DNA strand, and the pegRNA template is then used by the RT to directly synthesize the edited DNA sequence at the target site. Cellular processes then integrate the edited sequence.

The capacity to precisely alter the genetic code within living organisms marks a groundbreaking advancement in medicine [1, 2]. Such genome editing technologies present enormous potential for rectifying disease‐causing mutations, designing desirable traits and investigating core biological functions with exceptional precision [3]. Early strategies in this domain relied on engineered nucleases like zinc‐finger nucleases (ZFNs) and transcription activator‐like effector nucleases (TALENs) to create targeted DNA double‐strand breaks (DSBs) (Figure 1A,B) [7]. However, these pioneering methods were hindered by the need for complex protein engineering for every new target sequence, limiting their widespread use (Table 1). The field underwent a paradigm shift with the discovery and application of the bacterial CRISPR‐Cas system, especially the Cas9 nuclease derived from *Streptococcus pyogenes* (SpCas9) [8]. By employing a customizable guide RNA (gRNA) for DNA targeting, the CRISPR‐Cas9 system offered unprecedented simplicity, efficiency and versatility, which rapidly accelerated its adoption in a vast array of research and therapeutic settings (Figure 1C) [9].

**TABLE 1:** Comparative Overview of Key Genome Editing Technologies: ZFNs, TALENs, CRISPR‐Cas9, Base Editors and Prime Editors.

Major genome editing technologies	Molecular mechanism	Target recognition	Ease of design and use	Editing precision and efficiency	Off‐target activity	Common delivery methods	Clinical or research applications
Zinc Finger Nucleases (ZFNs)	Induces double‐strand breaks (DSBs) using engineered zinc finger proteins fused to a FokI nuclease domain. Requires dimerization of two ZFN units.	Engineered zinc finger arrays recognize 3‐bp DNA sequences (typically 9–18 bp total). Protein‐DNA interaction.	Difficult, expensive, time‐consuming (months). Requires protein engineering expertise. Limited target sites.	High specificity possible. Variable efficiency (1%–50% reported). DSB repair (NHEJ) causes indels. Precision via HDR.	Can occur; reduced by obligate heterodimers. Requires validation.	Viral vectors (Adeno, AAV, Lenti), plasmids, mRNA, protein.	Early gene therapy trials, cell line/animal generation. Less common now.
Transcription Activator‐Like Effector Nucleases (TALENs)	Induces DSBs using engineered TALE proteins fused to a FokI nuclease domain. Requires dimerization of two TALEN units.	Engineered TALE repeat arrays recognize single DNA bases (often 18+ bp total). Protein‐DNA interaction.	Easier than ZFNs (simpler code: 1 repeat = 1 base) but still requires complex protein assembly. Faster design than ZFNs (days/weeks). Fewer target site constraints.	High specificity (long recognition). Variable efficiency. DSB repair (NHEJ/HDR) causes indels or precise edits.	Generally lower off‐target than early Cas9 (longer target, dimerization). Requires validation.	Viral vectors, plasmids, mRNA. Larger than ZFNs.	Gene therapy research, disease modelling, GMOs (plants), functional genomics.
CRISPR‐Cas9	Induces DSBs using the Cas9 nuclease guided by a single guide RNA (sgRNA).	sgRNA base‐pairs (~20 nt) with target DNA adjacent to a Protospacer Adjacent Motif (PAM). RNA–DNA interaction.	Simple, fast (days), inexpensive design (sgRNA synthesis). Highly scalable and accessible.	High efficiency (> 70% possible) but varies. DSB repair prone to indels (NHEJ). Precise edits via HDR less efficient. High‐fidelity Cas9 improves precision.	Primary concern (DSBs at similar sites). Reduced by high‐fidelity Cas9, modified gRNAs, RNP delivery. Requires validation.	Viral vectors (AAV often dual), plasmids, mRNA, RNP electroporation, LNPs.	Basic research (functional genomics, screens), disease modelling, diagnostics, agriculture, gene therapy (approved therapies, e.g., SCD).
Base Editors (BEs)	Chemically converts a target DNA base (C>T or A>G) using a deaminase fused to a Cas9 nickase/dead Cas9, guided by an sgRNA. Avoids DSBs; may nick one strand.	sgRNA base‐pairs with target DNA near a PAM, guiding the Cas9‐deaminase fusion. RNA–DNA interaction.	Relatively simple design (sgRNA design). Requires selecting appropriate BE version for edit window.	High precision & efficiency for specific base conversions (C>T, A>G). Avoids DSBs & associated indels. Cleaner point mutation edits.	No DSB‐related off‐targets. Can have sgRNA‐dependent (off‐target sites) or independent (DNA/RNA) base conversions. Low indel rates. Requires validation.	Viral vectors, plasmids, mRNA, RNP, LNPs. Larger than Cas9.	Correcting pathogenic point mutations for gene therapy (research & pre‐clinical). Research tool for specific base changes.
Prime Editors (PEs)	Uses a Cas9 nickase fused to a reverse transcriptase (RT). Guided by a prime editing guide RNA (pegRNA) which includes an RT template. Nicks one DNA strand, reverse transcribes edit from pegRNA template onto target site. Avoids DSBs.	pegRNA base‐pairs with target DNA near a PAM. pegRNA also contains Primer Binding Site (PBS) and RT template sequence. RNA–DNA interaction.	More complex design than CRISPR/BEs due to pegRNA structure (spacer, scaffold, PBS, RT template). Often requires Optimisation for efficiency.	Highly versatile: all 12 base conversions, small insertions and deletions (~< 100 bp). High precision for intended edits. Efficiency varies greatly, often lower than BEs but improving. Avoids DSBs & indels.	Lower than Cas9 nuclease (no DSBs, multiple binding steps). Low indel rates. Potential for off‐target RT activity or pegRNA‐related off‐targets exists but often minimal. Requires validation.	Viral vectors (AAV), plasmids, mRNA, RNP, LNPs, eVLPs. Larger system size is a challenge for viral delivery.	Research: precise mutation modelling, correcting diverse mutations. Potential therapy for genetic diseases (point mutations, indels) not fixable by BEs (pre‐clinical).

*Note:* This table provides a detailed comparison of five major genome editing technologies: Zinc Finger Nucleases (ZFNs), Transcription Activator‐Like Effector Nucleases (TALENs), CRISPR‐Cas9, Base and Prime Editors. The comparison covers the following key aspects: Molecular mechanism, target recognition method, ease of design and use, editing precision and efficiency, off‐target activity, common delivery methods and clinical or research applications. The main purpose is to highlight the strengths, weaknesses and use cases of each technology in the context of genome editing applications.

### 1.2 Limitations of Nuclease‐Based CRISPR Editing

A key operational feature of the powerful CRISPR‐Cas9 system is its induction of DSBs at the targeted DNA site [10]. The cellular repair mechanisms that follow these breaks determine the editing outcome. In mammals, the main repair route, non‐homologous end joining (NHEJ), is error‐prone and typically generates random insertions or deletions (indels) [11], limiting its utility for precise editing, although effective for gene knockouts [12]. The alternative, homology‐directed repair (HDR), enables precise edits like single nucleotide corrections by using a donor DNA template [13]. However, HDR presents significant challenges due to its low efficiency compared to NHEJ, particularly in post‐mitotic cells and the complex requirement of delivering a donor template [14]. Critically, the DSBs central to CRISPR‐Cas9's function are not without risk; they can lead to unintended large deletions, chromosomal abnormalities and activation of the p53 damage response, potentially causing cell cycle arrest or apoptosis and thus posing safety concerns for clinical applications [5].

### 1.3 The Need for Precision: Emergence of Base Editing

The challenges associated with DSB‐dependent editing, particularly the unpredictable and inefficient nature of HDR for precise point mutation correction [15], combined with the fact that single nucleotide variants (SNVs) cause a large fraction of known human genetic diseases [16, 17, 18], spurred the advancement of more accurate gene editing tools [4, 19]. Base editing has recently surfaced as a revolutionary technology created to specifically tackle these challenges (Figure 1D) [20]. Introduced in 2016 by research groups including those led by David Liu and Akihiko Kondo, this technology allows for the direct, programmable chemical change of one DNA base pair to another (like C•G to T•A or A•T to G•C) [21]. A key advantage is that base editing functions without inducing DSBs and does not rely on HDR or donor DNA templates, leading to higher efficiency (particularly in non‐dividing cells) and fewer indel byproducts compared to traditional nucleases [14]. This development represents a significant step in the evolution of genome editing: from the protein engineering complexities of ZFNs/TALENs, to the RNA‐programmable but DSB‐reliant Cas9, and now to base editors capable of direct chemical modification without DSBs [22]. This evolution highlights a continuous drive towards safer, more efficient and precise genome editing technologies.

### 1.4 Scope and Aims of the Review

This paper provides an extensive overview of the rapidly evolving field of CRISPR‐based DNA base editing. It delves into the foundational discoveries behind cytosine and adenine base editors, clarifies their molecular operating principles and weighs their key strengths and weaknesses. The article highlights significant advancements that have boosted editor performance, broadened their targeting range and introduced new capabilities, including novel editors designed for transversion mutations, simultaneous dual‐base changes and editing within organelles or on RNA. Furthermore, the review examines diverse preclinical applications, such as creating disease models and performing functional genomic studies. It also surveys the current landscape of clinical translation, spotlighting prominent ongoing trials by companies like Verve Therapeutics and Beam Therapeutics. Finally, the discussion addresses major hurdles in in vivo delivery, explores the ethical dimensions surrounding this transformative technology and offers an outlook on future progress and therapeutic possibilities.

## 2 Discovery of Base Editing

### 2.1 Base Editing: Precision Genome Modification Without DSBs

Base editing was developed to circumvent the challenges associated with conventional CRISPR‐Cas9 editing, particularly for applications requiring precise single‐base corrections. The key innovation enabling base editing was to utilize the targeting function of CRISPR‐Cas9 without inducing DSBs or relying on HDR. This was achieved by transforming Cas9 from a nuclease into a precise DNA‐binding module. A catalytically impaired Cas9 (dCas9) or a nickase version (nCas9) was fused to a deaminase enzyme [23]. Upon guidance to the target locus by a gRNA, this engineered protein binds to the DNA, creating a localized region of single‐stranded DNA accessible to the fused deaminase, which then executes the intended base change chemically.

### 2.2 Cytosine Base Editors (CBEs): The First Breakthrough

CBEs, the first successful iteration of the base editing concept, enabled the specific conversion of C•G to T•A base pairs. This breakthrough was independently published in 2016 by the laboratories of David Liu (Harvard/Broad Institute) and Akihiko Kondo (Kobe University) [24, 25]. The initial base editor construct, Base Editor 1 (BE1), comprised the rat cytidine deaminase enzyme APOBEC1 linked to a dCas9 rendered nuclease‐dead by D10A and H840A mutations. When directed to a specific DNA target by a gRNA, BE1 binds, forming an R‐loop structure. Within the single‐stranded DNA exposed in this loop, the rAPOBEC1 enzyme catalysed the conversion of C to uracil U. BE1 was effective at C‐to‐U conversion, but its efficiency was markedly reduced in human cells [24].

To overcome this, BE2 was created by appending a potent UDG inhibitor peptide (UGI) to the C‐terminus of BE1, preventing the cellular machinery from excising the edited uracil. BE2 achieved approximately three times higher C•G to T•A editing efficiency while maintaining very low indel formation (≤ 0.1%). Further improvements led to BE3, where Cas9 was modified into a nickase (nCas9 D10A) to nick the non‐edited strand, favouring the edited strand during mismatch repair. BE3 significantly outperformed BE2, showing a 2‐ to 6‐fold increase in efficiency and generating fewer indels compared to the traditional Cas9 nuclease with HDR.

### 2.3 Adenine Base Editors (ABEs): Expanding the Toolkit

Although CBEs marked a major advance, they were limited to C•G to T•A conversions. Converting A•T to G•C, which accounts for about half of pathogenic point mutations, required a new approach [26, 27, 28]. David Liu's lab, led by Nicole Gaudelli, engineered the *E. coli* tRNA adenine deaminase (TadA) to act on single‐stranded DNA, using directed evolution techniques such as Phage‐Assisted Continuous Evolution (PACE) [29, 30, 31].

The resulting adenine base editors, exemplified by ABE7.10, fused an evolved TadA variant to nCas9 to mediate A•T to G•C conversions efficiently in human cells, achieving average editing efficiency around 53% with minimal indel formation (≤ 0.1%) [32]. Unlike CBEs, ABEs required the *de novo* evolution of an enzymatic function not naturally occurring in DNA, highlighting the innovative potential of directed evolution. Together with CBEs, ABEs now enable all four transition mutations (C•G to T•A, G•C to A•T, A•T to G•C and T•A to C•G), substantially expanding the range of correctable pathogenic point mutations via base editing.

## 3 Mechanism of Action

### 3.1 Core Components and Architecture

Base editors are specially engineered molecular tools composed of multiple protein domains, each performing a unique function and are guided to specific genomic targets by a gRNA molecule.

#### 3.1.1 DNA‐Binding Module (Cas9 Variant)

The foundation of most base editors is an engineered variant of the CRISPR‐Cas9 protein, typically derived from *Streptococcus pyogenes* . However, unlike the fully active nuclease employed in conventional CRISPR editing, base editors incorporate Cas9 versions with modified catalytic activity [27]. Early versions of base editors, such as BE1 and BE2, employed a dCas9 that harboured mutations (D10A and H840A) disabling both of its nuclease domains. This modification enabled the dCas9 to bind target DNA, as directed by a gRNA, without introducing DSBs [24].

In contrast, subsequent and contemporary base editors, such as BE3, BE4 and ABEs, predominantly incorporate an nCas9, which cuts only one DNA strand [3]. A widely used nCas9 variant carries the D10A mutation, which inactivates the RuvC nuclease domain tasked with cutting the non‐target DNA strand. Meanwhile, the HNH domain remains active and introduces a single‐strand nick in the target strand that is complementary to the gRNA [28]. Alternatively, the H840A mutation can inactivate the HNH nuclease domain, generating a Cas9 nickase that cleaves only the non‐target DNA strand. However, most base editing systems commonly employ the D10A nickase variant.

Regardless of whether dCas9 or nCas9 is used, the primary role of the Cas9 component in a base editor is to serve as a programmable DNA‐binding scaffold, precisely guiding the fused deaminase enzyme to the genomic locus specified by the gRNA sequence. Furthermore, in base editing systems that employ a nCas9, the resulting single‐strand break (nick) is thought to influence cellular DNA repair pathways, biasing them towards resolving the mismatch in favour of incorporating the intended base change on the opposing strand.

#### 3.1.2 Targeting Module (gRNA)

Specificity within the CRISPR‐Cas9 machinery is provided by the sgRNA. This synthetic RNA molecule combines essential features from the natural CRISPR RNA (crRNA) and trans‐activating crRNA (tracrRNA). Its 5′ end contains a variable ‘spacer’ region of about 20 nucleotides, designed to match the target DNA sequence (protospacer). The sgRNA directs the Cas9‐deaminase fusion protein to the target genomic site through base pairing between the spacer and protospacer [33]. Successful targeting also necessitates a short, conserved DNA sequence called the protospacer adjacent motif (PAM), situated immediately next to the protospacer site. While the standard SpCas9 typically requires an NGG PAM sequence (N being any base), engineered Cas9 variants have been developed with different PAM specificities, increasing the number of potential target sites throughout the genome [34].

#### 3.1.3 Catalytic Module (Deaminase Enzyme)

The deaminase component functions as the catalytic engine of the base editor, performing the chemical transformation on the targeted nucleotide base. As a result, the type of base conversion that occurs is determined by the identity of the fused deaminase. Specifically, CBEs employ cytidine deaminase enzymes to achieve the desired base modification [35]. These enzymes function by catalysing the hydrolytic deamination of a cytosine base, effectively converting it into uracil. While the rat‐derived APOBEC1 is the most frequently utilized deaminase in CBEs, other enzymes such as human APOBEC3A (hA3A) [36], lamprey PmCDA1 [37] and human activation‐induced cytidine deaminase (AID) [15] have also been incorporated or further engineered to potentially improve CBE performance characteristics.

ABEs require a DNA adenine deaminase, an enzyme not found naturally. To overcome this, scientists engineered versions based on the TadA [38]. The initial breakthrough yielded the TadA‐7.10 variant, commonly employed as a heterodimer with wild‐type TadA within the ABE7.10 editor system. Further directed evolution efforts have since produced enhanced versions with improved properties, such as the TadA‐8e variant utilized in the ABE8e editor. These evolved TadA enzymes catalyse the deamination of adenine within the DNA substrate, converting it to inosine [5].

UGI functions as a key repair module in most CBEs, especially those developed from BE2 onwards, significantly boosting their efficiency and specificity [39]. UGI is a small protein from the PBS1 bacteriophage that potently blocks the cell's UDG enzyme [40]. Normally, UDG initiates base excision repair (BER) by identifying and removing uracil from DNA [41]. By inhibiting UDG, the UGI component prevents the excision of the uracil base produced by the CBE's deaminase activity. Protecting this uracil intermediate greatly increases the probability that the resulting U•G mismatch is converted into the desired T•A base pair through cellular DNA replication or repair. Because the inosine (I) generated by ABEs is not efficiently recognized and removed by cellular DNA glycosylases, unlike the uracil produced by CBEs, ABEs typically do not require UGI.

### 3.2 Molecular Steps of Base Editing

**FIGURE 2:**
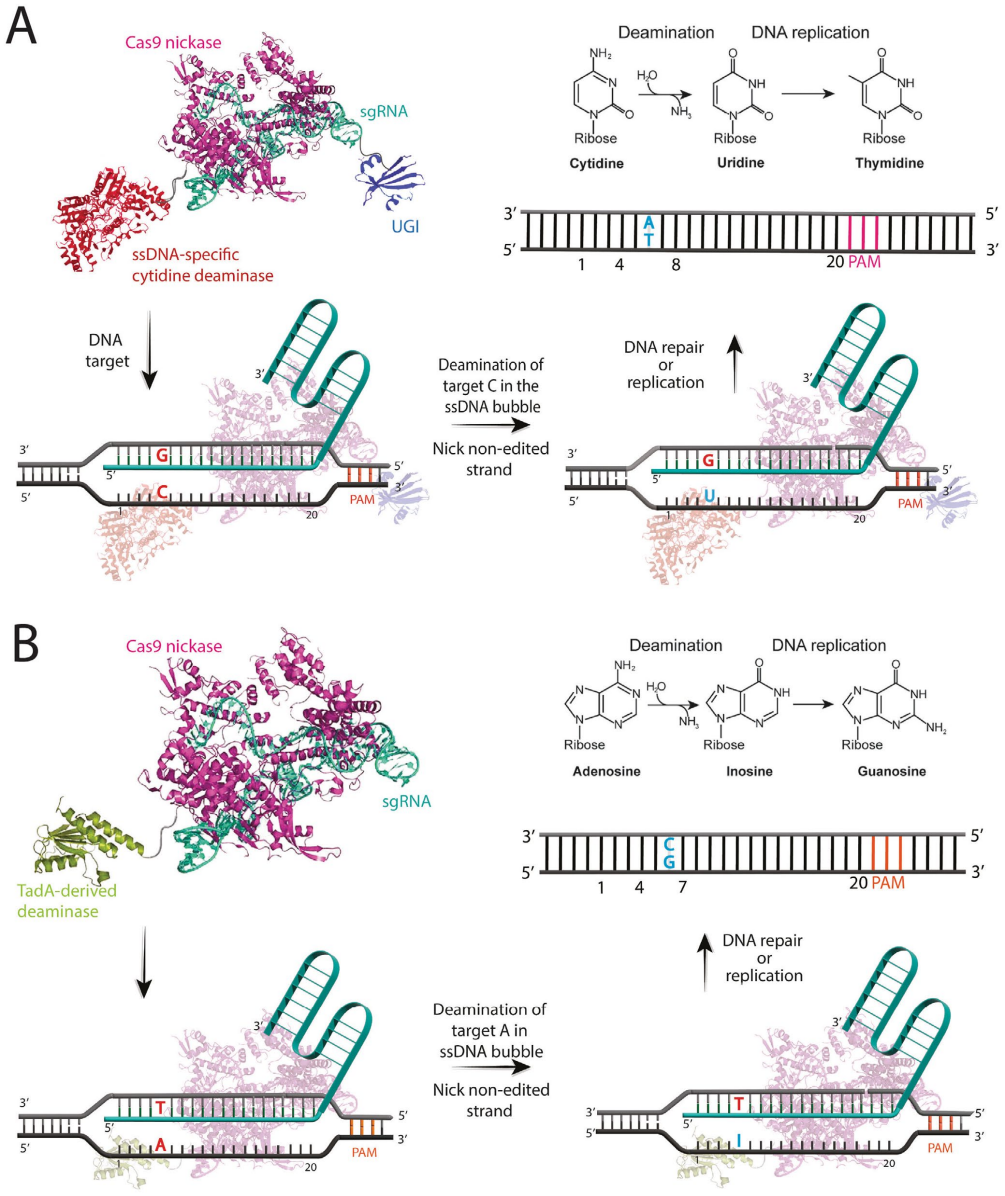
Schematic Representation of Base Editing Using CRISPR‐Derived Base Editors [31, 42]. Base editors are engineered fusion proteins consisting of a catalytically impaired Cas9 enzyme either a nickase variant (nCas9), which introduces a single‐strand cut, or a catalytically inactive variant (dCas9), which lacks nuclease activity linked to a DNA deaminase enzyme [24]. Guided by a sequence‐specific sgRNA, the base editor complex localizes precisely to the target DNA sequence, where the gRNA hybridizes with the target DNA strand to form an RNA–DNA heteroduplex. This process creates an R‐loop structure that displaces the non‐target strand, leaving it single‐stranded and accessible to the fused deaminase. Within a defined editing window on this displaced non‐target strand, the deaminase catalyzes the chemical conversion of a specific nucleotide: Cytosine is deaminated to uracil (read as thymine during DNA replication), enabling C•G to T•A transitions (Panel A), while adenine is deaminated to inosine (interpreted as guanine), allowing A•T to G•C transitions (Panel B). In constructs utilizing nCas9, a nick is introduced on the target (non‐edited) DNA strand to promote repair mechanisms that favor the incorporation of the desired edit, enhancing editing efficiency. This approach allows highly efficient and accurate single‐base substitutions while minimizing the risks of double‐strand breaks, insertions, deletions and other unintended genomic alterations.

Base editing involves a precise sequence of molecular events orchestrated between the engineered editor tool and the cell's own systems (Figure 2). First, the base editor complex, which includes a modified Cas9 protein, a deaminase enzyme (plus UGI for CBEs) and a gRNA, scans the DNA for a specific sequence called a PAM. After recognizing the PAM, the Cas9 protein attaches to the neighbouring DNA and begins unwinding the double helix [33]. If the DNA sequence next to the PAM matches the gRNA, the gRNA locks onto its target DNA strand, pushing the other DNA strand aside and forming an ‘R‐loop’. During R‐loop formation, the gRNA base‐pairs with the target DNA strand, creating an RNA–DNA hybrid and consequently displacing the non‐target strand. This displaced non‐target strand becomes single‐stranded and accessible, held open by the Cas9‐gRNA structure [5]. This exposed single strand within the R‐loop allows the attached deaminase enzyme to perform its function [43].

The deaminase chemically changes a specific base (cytosine for CBEs, adenine for ABEs) located within a defined ‘editing window’ inside the loop, with the precise location and size (often spanning a few bases, like positions 4–8) determined by the specific editor's design [28]. CBEs convert C to U, creating a U•G base mismatch, while ABEs use an engineered TadA deaminase to convert A to inosine I, which cellular machinery reads as G, thus forming an I•T mismatch.

Once a CBE creates a U•G mismatch, the cell recognizes this pairing as incorrect. In CBE versions equipped with UGI (like BE2, BE3, BE4), this inhibitor prevents the enzyme UDG from starting the usual BER pathway. Because BER is blocked, the cell relies on other systems, mainly MMR or the natural process of DNA replication, to resolve the mismatch. When DNA replication processes the U•G mismatch, the uracil acts as a template, causing an adenine to be incorporated into the new strand. Consequently, one daughter DNA molecule ends up with a T•A pair, successfully converting the original C•G site (following the C•G → U•G → T•A). For CBEs designed as nickases (e.g., BE3, BE4), a break is made on the opposite strand (containing the guanine). This nick encourages the MMR system to use the uracil‐containing strand as the correct template, replacing the guanine on the nicked strand with an adenine. This creates a U•A pair, which becomes the desired T•A pair after the next DNA replication cycle.

Inosine (I), generated by ABEs, isn't a standard DNA base. However, because its chemical structure is very similar to guanine, the cell's DNA polymerase enzymes treat it like guanine during replication and repair. Consequently, when the replication machinery encounters an inosine base on the template strand, it typically inserts a cytosine into the new complementary strand. This effectively converts the initial A•T target pair first into an I•T mismatch, then into an I•C pair during the processing of the complementary strand. Ultimately, after subsequent DNA replication, this sequence results in a stable G•C base pair in the daughter DNA molecules. Additionally, the nick created by the nCas9 component on the non‐edited, thymine‐containing strand is believed to further encourage repair mechanisms to use the inosine‐containing strand as the template, reinforcing the desired edit [33].

Successful base editing arises from a crucial interdependence between the engineered editor tool and the host cell's own DNA processing machinery. The editor itself handles targeting the specific DNA site and making the initial chemical change to the base. This process fundamentally hijacks the cell's natural replication and repair systems to permanently alter it. Because base editing harnesses internal cellular processes, its efficiency and precision vary depending on the specific cellular environment. Key factors influencing the final edit include cell type, cell cycle stage and the activity of DNA repair pathways. For instance, studies suggest that editors equipped with a nickase function operate consistently across the cell cycle, whereas non‐nickase editors might achieve optimal results during the S‐phase concurrent with DNA replication [44]. Furthermore, experiments performing cytosine base editing in synchronized cells revealed that editing during the G1 phase can increase certain types of unwanted mutations, implying that the availability and balance of repair pathways are likely to change throughout the cell cycle, influencing the editing results. This context‐dependent behaviour highlights that base editors are not just chemical agents but sophisticated biological tools that interact dynamically with the cellular environment.

## 4 Performance Trade‐Offs and Engineering Strategies in Base Editing

As a major advancement in CRISPR‐Cas technology, base editing offers not only distinct benefits for specific genetic engineering applications but also presents its own unique difficulties (Table 1) [14].

### 4.1 Advantages Over Nuclease‐Based Editing

A major benefit of base editors lies in their capability to directly and accurately change specific single DNA base pairs right at the target location [3]. This makes them exceptionally suitable for correcting many genetic diseases caused by such point mutations. Crucially, base editors alter DNA without cutting both strands, unlike conventional CRISPR‐Cas9. By avoiding DSBs, base editing circumvents associated risks like large DNA segment deletions, chromosomal rearrangements, translocations and potential cell toxicity linked to the p53 response [5]. As a result, base editing typically leads to substantially fewer unwanted indels than conventional Cas9 nuclease editing. This results in more predictable and cleaner editing at the target site.

Unlike precise editing with Cas9 that uses HDR, base editing achieves its result without needing an externally supplied DNA repair template. This makes the task of getting the editing machinery into cells easier, which could lead to a less complex and more cost‐effective process. Base editing is effective even in cells that don't divide or divide only rarely, such as neurons and terminally differentiated cells. This is because it does not depend on the HDR pathway, which is predominantly active only during specific phases (S and G2) of the cell division cycle. This feature greatly increases the number of tissues and cell types that are receptive to precise gene correction, unlike strategies requiring HDR.

### 4.2 Specificity Challenges: Off‐Target DNA and RNA Editing

Although powerful, base editing technologies have drawbacks and potential hazards, needing careful assessment, especially before clinical use. A primary concern is unintended edits occurring at locations away from the intended target site, known as off‐target DNA editing. This can happen in two main ways. Firstly, like standard CRISPR, the gRNA directs the base editor, but if the specificity isn't perfect, editing can occur at sites with similar sequences. These unintended point mutations, even without DSBs, pose a safety risk. Strategies to lessen this include using high‐fidelity Cas9 components and carefully designing the gRNAs [36]. Secondly, off‐target editing can occur independently of the Cas enzyme binding, particularly with CBEs that use APOBEC‐family deaminases. In these systems, the deaminase component itself might modify vulnerable single‐stranded DNA regions anywhere in the genome, not just where the Cas9 is bound [5]. This could lead to widespread C‐to‐T changes, especially within active genes. While ABEs exhibit less of this activity, it remains a significant safety consideration. Possible solutions include modifying the deaminase enzyme for greater precision or shortening the duration of the editor's activity within the cell, for instance, by using temporary delivery techniques like ribonucleoprotein (RNP) electroporation [36].

A key challenge with base editors (both CBEs and ABEs) is that their deaminase enzymes can unintentionally modify RNA molecules throughout the cell, in addition to their intended DNA targets. This results in widespread, guide‐RNA‐independent off‐target edits (C‐to‐U by CBEs, A‐to‐I by ABEs) in the transcriptome [5]. Although these RNA edits are transient because RNA degrades, their potential effects on cell function raise safety concerns as they are not fully understood. To address these issues, extensive engineering efforts have focused on improving the specificity of base editors (Table 2). Incorporation of high‐fidelity Cas9 variants such as SpCas9‐HF1 has been shown to substantially reduce guide‐dependent off‐target DNA editing. For example, the HF‐BE3 editor uses SpCas9‐HF1 to lessen off‐target DNA editing arising from non‐specific Cas9 binding, even though this can occasionally result in slightly decreased efficiency at the desired target site [45].

**TABLE 2:** Summary of Cytosine, Adenine, Dual and Glycosylase‐Based Base Editor Variants and Their Key Properties.

Editor name	Key components	PAM (Common)	Editing window (approx. pos.)	Notes on efficiency/fidelity
Cytosine Base Editors (CBEs)				
BE1	rAPOBEC1 + dCas9 (D10A + H840A)	NGG	(4–8)	First generation, low efficiency. Uses dead Cas9 (dCas9).
BE2	rAPOBEC1 + dCas9 + UGI	NGG	(4–8)	Added Uracil Glycosylase Inhibitor (UGI) to increase efficiency over BE1.
BE3	rAPOBEC1 + nCas9(D10A) + UGI	NGG	(4–8)	Uses Cas9 nickase (nCas9) instead of dCas9, further boosting efficiency. Can cause indels.
HF‐BE3	rAPOBEC1 + HF‐ nCas9 (D10A) + UGI	NGG	(4–8)	Uses High‐Fidelity SpCas9 variant (HF1) to reduce off‐target DNA editing.
YE1‐BE3	rAPOBEC1(W90Y + R126E) + nCas9(D10A) + UGI	NGG	(5–6)	BE3‐like maximal editing efficiencies, but narrowed editing window width.
EE‐BE3	rAPOBEC1(R126E + R132E) + nCas9(D10A) + UGI	NGG	(5–6)	Incorporates dual mutations that suppress DNA and RNA off‐target editing, but reduces on‐target DNA editing efficiency.
YE2‐BE3	rAPOBEC1(W90Y + R132E) + nCas9(D10A) + UGI	NGG	(5–6)	Similar editing efficiencies as EE‐BE3, lower than wide‐type BE3, but with narrowed editing window.
YEE‐BE3	rAPOBEC1(W90Y + R126E + R132E) + nCas9(D10A) + UGI	NGG	(5–6)	Exhibits 2.9‐fold lower average editing compared to BE3 but very little editing beyond the C6 position.
VQR‐BE3	rAPOBEC1 + VQR nCas9 (D10A, D1135V, R1335Q, T1337R) + UGI	NGAN	(4–8)	Employs the Cas9‐VQR variant to expand PAM compatibility to NGA, while maintaining C to T base editing activity similar to BE3.
EQR‐BE3	rAPOBEC1 + EQR nCas9 (D10A, D1135E, R1335Q, T1337R) + UGI	NGAG	(4–8)	Uses the Cas9‐EQR variant to enable recognition of NGAG PAMs, demonstrating efficient C to T editing with on‐target activity comparable to VQR‐BE3.
VRER‐BE3	rAPOBEC1 + VRER nCas9 (D10A, D1135V, G1218R, R1335E, T1337R) + UGI	NGCG	(4–8)	Incorporates the Cas9‐VRER variant, conferring recognition of NGCG PAMs, though with lower efficiency relative to BE3.
SaKKH‐BE3	rAPOBEC1 + SaKKH nCas9 (D10A, E782K, N968K, R1015H) + UGI	NNNRRT	(3–12)	Utilizes the engineered SaCas9‐KKH variant to expand PAM compatibility, thereby broadening the targeting scope of C to T base editing, efficiency up to 60%.
SECURE‐BE3	nCas9(D10A) + rAPOBEC1 (R33A, R33A/K34A) + UGI	NGG	(5–7)	Uses modified APOBEC1 deaminase to reduce unwanted RNA off‐target activity while retaining high on‐target DNA editing efficiency.
xBE3	rAPOBEC1 + nxCas9(D10A) + UGI	NG	(4–8)	Uses xCas9 variant with broader NG PAM compatibility.
BE4	rAPOBEC1 + nCas9(D10A) + 2 × UGI	NGG	(4–8)	Added second UGI and optimized linkers; improved product purity (less C>G/C>A) vs. BE3.
BE4‐Gam	BE4 + Gam (from Mu phage)	NGG	(4–8)	Fused Gam protein reduces indel formation associated with nickase activity.
SaBE3/SaBE4	rAPOBEC1 + nSaCas9 + UGI (or 2 × UGI)	NNGRRT	(3–12)	Uses smaller *Staphylococcus aureus* Cas9 (SaCas9) for packaging (e.g., in AAV).
BE4max/AncBE4max	rAPOBEC1 (or Anc) + nCas9(D10A) + 2 × UGI	NGG	(4–8)	Codon Optimisation and NLS addition (BE4max) or ancestral deaminase reconstruction (AncBE4max) for higher efficiency.
evoAPOBEC1‐BE4max	evoAPOBEC1 + nCas9(D10A) + 2 × UGI	NGG	4–8 (better GC)	Evolved deaminase improves editing efficiency in GC sequence contexts.
evoCDA1‐BE4max	evoCDA1 + nCas9(D10A) + 2 × UGI	NGG	Broader (1–13)	Evolved PmCDA1 deaminase provides a much wider editing window.
evoFERNY‐BE4max	evoFERNY + nCas9(D10A) + 2 × UGI	NGG	4–8 (better GC)	Uses smaller cytodine deaminase, facilitating easier fusion to Cas9 and improving delivery efficiency.
A3A(N57G)‐BE4max	hA3A(N57G) + nCas9(D10A) + 2 × UGI	NGG	Broader (1–17)	Uses human APOBEC3A variant for broad window; N57G mutation increases fidelity (less C>G/A).
SpRY‐CBE4max	CBE4max + SpRY nCas9	NRN/NYN	(4–8)	Near PAM‐less SpCas9 variant (SpRY) fused to CBE4max for broad targeting range.
Target‐AID	PmCDA1 + dCas9 + UGI	NGG	(2–4)	Uses deaminase from sea lamprey (PmCDA1). Different editing window/profile than BE3.
BE‐PLUS	rAPOBEC1 + scFv + nCas9(D10A) + 10xGCN4 + UGI	NGG	4–16 (better GC)	Uses antibody fragment (scFv) to recruit UNG inhibitor, expanding the editing window.
LbCas12a‐BE	rAPOBEC1 + dLbCas12a + UGI	TTTV	Downstream (8–13)	Uses Cas12a (Cpf1) for T‐rich PAMs. Editing window downstream of guide (V is A, C, or G).
CE_CBEs	APOBEC3A‐ nCas9 + UGI	NGG	(4–8)	Cytidine deaminase fused into the Cas9 protein reduces off‐target effects and improves the stability and solubility of the base editor.
CGBE1	rAPOBEC1(R33A) + nCas9(D10A) + eUNG	NGG	(4–8)	First C•G to G•C editor. Fuses engineered UNG from *E. coli* to convert U to an intermediate, with higher C to G editing activity.
Tad‐CGBE	Engineered TadA* + nCas9(D10A) + UNG or TDG	NGG	(4–8)	Uses TadA variants to achieve highly efficient and precise C•G to G•C editing.
TadCBE	Engineered TadA* + nCas9(D10A) + 2 × UGI	NGG	Variable	Uses engineered adenine deaminase TadA* variants for C>T editing. Often smaller size, high fidelity.
DdCBEs	DddA + TALE + UGI	None	(4–6)	Split DddA cytidine deaminase to achieve C•G‐to‐T•A editing in mtDNA. Unlike CBEs, DdCBEs induce C to T editing on both DNA strands.
RESCUE	ADAR2 + dCas13 + gRNA	None	Depends on the protospacer	Enables transient, programmable C to U editing in RNA without altering DNA. Editing efficiency might be up to 42%.
Adenine Base Editors (ABEs)				
ABE7.10	TadA‐TadA*(7.10) + nCas9(D10A)	NGG	(4–7)	First efficient ABE. Uses engineered *E. coli* TadA dimer. Generally high fidelity, low indels.
ABEmax	TadA‐TadA*(7.10) + nCas9(D10A)	NGG	(4–7)	Optimized expression (ABEmax) for higher efficiency.
SaABE	TadA‐TadA*(7.10) + nSaCas9	NNGRRT	(4–14)	ABE fused to smaller SaCas9.
xABE	TadA‐TadA*(7.10) + nxCas9(D10A)	NG	(4–7)	ABE fused to xCas9 for broader NG PAM compatibility.
SECURE‐ABE	TadA7.10 (K20A + R21A) + nCas9(D10A)	NGG	(4–7)	Engineered TadA variants reduce guide‐independent RNA off‐target editing while maintaining efficient A to G editing.
ABE8e/ABE8s	TadA*(8e/8 s) + nCas9(D10A)	NGG	4–8/3–9 (wider peak)	Highly efficient 8th gen ABEs. Much faster kinetics. ABE8e may increase bystander edits vs. ABE7.10.
Nme2‐ABE8e	TadA*(8e) + nNme2Cas9	NNNNCC	Variable	Uses *Neisseria meningitidis* Cas9 for different PAM and potentially higher fidelity.
NG‐ABE9e	TadA*(9e) + nCas9(D10A)	NG	4–7 (narrower peak)	Engineered from ABE8e for reduced bystander editing while maintaining high on‐target efficiency.
ABE10	ABE8e with TadA‐8e A48E	NGG	4–8/3–9	Edits adenine within a ‘YAC’ sequence context, reducing unwanted edits at sites lacking this motif.
ABEmax‐AW	TadA(E59A)‐TadA7.10 (V106W) + nCas9(D10A)	NGG	(4–7)	Mutations introduced into both TadAs reduce unintended RNA editing while preserving high DNA on‐target activity, and minimizing indel by‐product formation.
ABE‐Umax	Optimized high‐activity TadA variant + nCas9(D10A)	NGG	(4–8)	Achieves high efficiency with low indel rates compared to ABEmax, offering flexible PAMs with reduced bystander mutations.
VRER‐ABEmax	TadA‐TadA*(7.10) + VRER nCas9	NGCG	(4–7)	Allows precise A to G editing at NGCG sites, with higher efficiency than ABEmax.
VRQR‐ABEmax	TadA‐TadA*(7.10) + VRQR nCas9	NGA	(4–7)	Uses the SpCas9‐VRQR variant to expand PAM recognition, enabling efficient A to G editing with high on‐target activity and low indel formation.
SaKKH‐ABEmax	TadA‐TadA*(7.10) + SaKKH nCas9	NNNRRT	(4–14)	Employs the engineered SaCas9‐KKH variant for NNNRRT PAM recognition, achieving 26% A•T‐to‐G•C conversion with minimal indels.
SpRY‐ABEmax	ABEmax + SpRY nCas9	NRN/NYN	(4–7)	Near PAM‐less SpCas9 variant (SpRY) fused to ABE for broad targeting range.
LbCas12a‐ABE	TadA‐TadA*(7.10) + nLbCas12a	TTTV	Downstream (14–17)	ABE fused to Cas12a (Cpf1) for T‐rich PAMs. Editing window downstream of guide (V is A, C, or G).
TurboABE	TadA‐TadA*(8e) + nCas9‐Rad51DBD	NGG	4–7 (improved low)	Incorporates Rad51 DNA‐binding domain to enhance editing at less favourable positions within window.
AYBE	TadA*(8e) + nCas9 (D10A) + MPG or AAG	NGG	(4–8)	Wild‐type human N‐methylpurine DNA glycosylase protein (MPG) or Alkyladenine DNA glycosylase (AAG) fused to ABE8e editor to convert A•T pairs to T•A or C•G.
mTALED/dTALED	DddA(E1347A) + TadA*(8e) + TALE/DddA(E1347A) + TadA*(8e) + 2 × TALE	None	Depends on the protospacer	Integrates TadA variants into TALE‐based framework to perform A•T‐to‐G•C edits in mtDNA. Effective editing within a 10 to 20 bp window located immediately downstream of the TALE protein's binding site on the mtDNA.
REPAIR	ADAR2 + dCas13	None	Depends on the protospacer	Programmable A to I editing without PAM constraints, providing transient, reversible, and precise RNA modification.
Dual Base Editors				
TadDE	Engineered TadA* + nCas9(D10A) + 2 × UGI	NGG	Variable	Uses engineered adenine deaminase TadA* variants for dual base editing. Often smaller size, high fidelity.
A&C‐BEmax	ABEmax + CBE4max	NGG	2–17 (both)	Dual editor capable of both C>T and A>G editing simultaneously using fused deaminases.
SPACE	ABEmax + PmCDA1+ 2 × UGI	NGG	4–7/2–7	Similar dual editor concept to A&C‐BEmax.
Target‐ACE	TadA‐TadA*(7.10) + nCas9(D10A) + PmCDA1 + UGI	NGG	1–8/4–8	Performs both C → T and A → G editing simultaneously.
STEMEs	hAPOBEC3A + ABE7.10 + nCas9 + UGI	NGG	C: 1 to 17 A: 4 to 8	Dual editor capable of both C to T and A to G editing simultaneously using fused deaminases.
pDuBE1	TadA‐8e + nCas9 (D10A) + eCDAL + UGI	NGG	C: 1 to 10 A: 4 to 11	Uses an eCDAL (enhanced CDA1‐like deaminase) derived from Japanese lamprey CDA1‐like 4 for highly efficient C to T editing.
Glycosylase‐based Base Editors				
gCBE	nCas9 + engineered human uracil DNA glycosylase (UNG)	NGG (varies)	(2–9)	Deaminase‐free gCBE enables C•G to G•C transversions; editing efficiencies up to ~77.7%, with possible indel formation.
gTBE	nCas9 + thymine DNA glycosylase (TDG)	NGG	(2–6)	Enables T•A to G•C transversions through TDG‐mediated thymine excision and subsequent AP‐site repair (~22% average efficiency; up to ~48%).
gGBE	nCas9 + an engineered N‐methylpurine DNA glycosylase (MPG)	NGG	(4–8)	Performs G to C transversions and induces indels as a byproduct of abasic site generation, thereby reducing overall fidelity.

*Note:* This table provides a comprehensive overview of various cytosine and adenine base editor variants, detailing their key properties, including PAM compatibility, editing window, editing efficiency and fidelity as of March 2026.

To address off‐target effects from deaminase activity, specific mutations have been introduced into the deaminase domains. In CBEs, SECURE (SElective Curbing of Unwanted RNA Editing)‐CBEs variants (e.g., with R33A/K34A mutations in rAPOBEC1) significantly reduce the unwanted RNA editing while maintaining DNA editing activity [36]. Similarly, specific mutations in the ABE's TadA deaminase (e.g., V106W in ABEmaxAW; or E59A, D53E, F148A) substantially reduce off‐target RNA edits. Utilizing different deaminase enzymes offers another path to improving specificity. Engineered human APOBEC3A (hA3A) variants show potential for reducing bystander DNA edits, and further modifications (e.g., R128A, Y130F) can lower RNA off‐target activity. Additionally, new CBEs based on engineered TadA deaminases (Td‐CBEs) demonstrate improved off‐target profiles compared to traditional APOBEC‐based CBEs [46]. Researchers have also investigated structural changes to base editors. For example, Cas‐embedded CBEs (CE‐CBEs) insert the deaminase enzyme within the Cas9 protein sequence, which may improve solubility, enhance stability for RNP delivery and reduce unintended edits [39].

### 4.3 Precision Versus Editing Window Constraints

The deaminase component of a base editor acts within a defined stretch of DNA known as the ‘editing window,’ which typically covers several nucleotides. A potential issue arises if multiple bases susceptible to editing exist within this window at the target locus. In such cases, the deaminase might unintentionally modify these adjacent ‘bystander’ bases along with the intended target base [47]. These unintended edits can lead to unwanted consequences, such as changes in the resulting protein's amino acid sequence, which reduces the precision of the desired modification. Researchers have engineered base editors with modified editing windows to increase precision and minimize unwanted bystander mutations when multiple editable bases are near the target. Mutations in the rAPOBEC1 deaminase yielded CBE variants (e.g., YE1‐BE3, YEE‐BE3) featuring narrower editing windows (down to 2–3 bases) or shifted activity profiles, enhancing precision [48]. Editors like evoAPOBEC1‐BE4max had evolved specifically for efficiency in GC‐rich contexts [42]. ABE variants exhibit diverse window changes; ABE8r has an expanded PAM‐distal window, while suites like ABE‐Umax (for zebrafish) provide options with shifted, narrowed or broadened windows [49, 50]. Other modifications aim to increase editing precision; ABE10 (also known as ABE8e A48E) preferentially edits adenine within a ‘YAC’ sequence context, which helps reduce unwanted edits at sites lacking this motif [51]. Additionally, researchers have developed smaller deaminase components, like evoFERNY, to overcome related challenges associated with base editing [42].

While base editing generally provides precise outcomes, it is not always perfectly clean, and unintended genetic alterations can sometimes occur. For example, CBEs occasionally generate undesired by‐product mutations such as C•G to G•C or C•G to A•T transversions, instead of the intended C•G to T•A transition. This is hypothesized to occur when the UDG enzyme's activity is not fully blocked by the UGI, allowing processing by error‐prone Translesion Synthesis (TLS) polymerases [5]. Furthermore, although indel frequencies are typically much lower than with traditional nuclease‐based editing, they are not entirely absent and can vary depending on the specific editor, target DNA sequence and cell type used.

In many therapeutic contexts, perfect single‐nucleotide correction may not be strictly required, as therapeutic benefit can also be achieved through gene disruption or regulatory element modification. However, in clinical scenarios requiring precise correction of a specific pathogenic nucleotide, base editors may still face challenges in achieving single‐base accuracy. In such cases, prime editing has been proposed as a more precise genome editing strategy capable of introducing defined nucleotide substitutions without relying on an activity window [52]. Nevertheless, prime editing also presents its own technical limitations, including lower editing efficiencies in some cellular contexts and more complex delivery requirements, particularly for in vivo therapeutic applications.

### 4.4 PAM Constraints Versus Targeting Scope

The range of genomic sites that base editors can modify is restricted by the PAM specificity of their Cas9 component [11]. The widely utilized SpCas9 variant necessitates an NGG PAM sequence adjacent to the DNA target. This dependency limits editing capabilities to only those sequences located next to an NGG motif. Therefore, the inability to target sequences lacking the appropriate nearby PAM restricts comprehensive genome access.

To move beyond the targeting restrictions of the standard NGG PAM required by SpCas9, researchers have developed base editors using alternative Cas9 orthologs or engineered variants with modified PAM specificities [53]. By employing Cas9 versions that recognize simpler PAMs like NG (e.g., SpCas9‐NG), NGN (e.g., SpG‐Cas9) or those with highly relaxed requirements like NRN/NRH/NRA (e.g., SpRY‐Cas9, where *R* = A/G, H = A/C/T), the number of accessible target sites across the genome is significantly increased [34, 54]. Early PAM‐flexible base editors included engineered SpCas9 variants such as VQR‐BE3 (recognizing NGA PAMs), EQR‐BE3 (NGAG) and VRER‐BE3 (NGCG), which enabled cytosine editing at otherwise inaccessible loci [48]. Building upon this, base editing systems developed with next‐generation variants, including SpG‐BE, SpRY‐BE, NG‐ABEmax, SaKKH‐ABEmax and PhieABEs (using nCas9‐NG), substantially expand the portion of the genome amenable to base editing [23]. Moreover, ABEs adapted to altered PAM variants, such as VRER‐ABEmax and VRQR‐ABEmax, demonstrated efficient A•T‐to‐G•C conversion across multiple targets, with VRQR‐ABEmax having ~35% editing efficiency at NGA sites with minimal indel formation, while VRER‐ABEmax showed comparable activity across NGCG PAM sites [55]. Notably, with near‐PAMless variants (NRN (where R is A or G)) such as SpRY, it's projected that up to 95% of known pathogenic point mutations could potentially be targeted [56].

### 4.5 Efficiency–Safety Trade‐Offs in Base Editor Optimisation

Although base editing is often more precise than nuclease‐based editing, its efficiency can vary substantially across targets and cell types. Improving catalytic activity has therefore been a major focus of editor development. Within the CBE family, optimizing structural elements like inter‐domain linkers, codon usage and adding a second UGI unit improved efficiency and purity, as seen in BE4 and BE4max [28]. For ABEs, optimizing nuclear localization signals (NLS) and codons produced the highly active ABEmax. Subsequently, directed evolution of the TadA deaminase led to the hyper‐efficient TadA8e variant (in ABE8e), which works hundreds of times faster than the ABE7.10 version, significantly increasing editing rates [57]. Other powerful variants like ABE8r, evoCDA1‐BE4max and ABE‐Umax continue to expand the limits of editing efficiency [42, 49, 50].

However, improvements in catalytic efficiency do not always translate into uniformly improved therapeutic performance. Hyperactive editors may increase the risk of bystander editing or off‐target activity, and highly optimized systems can still exhibit strong locus dependence. Thus, editor development has followed a recurring pattern in which gains in efficiency, targeting flexibility or activity must be balanced against safety and precision. The historical progression from early editors such as BE1‐BE3 and ABE7.10 to higher‐fidelity and more specialized variants reflects this iterative balancing process [58, 59, 60].

### 4.6 Immunogenicity, Clinical and Ethical Limitations

A significant consideration for base editing therapies is immunogenicity, the potential to trigger an unwanted immune response. Because CRISPR‐Cas9 proteins originate from bacteria, the human immune system may recognize them as foreign and react against them [61]. Similarly, viral vectors frequently used to deliver the editing machinery can also provoke immune reactions [62]. Delivery methods that avoid viruses, like using lipid nanoparticles (LNPs) to carry mRNA or RNP forms of the editor, are generally considered less likely to cause these immune issues [63].

Beyond these technical challenges, an additional limitation for the clinical implementation of base editing arises from the extensive genetic heterogeneity underlying many human diseases. Although base editors are particularly effective for correcting defined point mutations, the majority of pathogenic variants occur sporadically and affect only a small number of patients. Evidence from large‐scale genomic cohorts reveals that 99% of human variants are found at low frequencies; consequently, much of the genetic landscape is composed of rare or patient‐specific mutations rather than common polymorphisms [64]. Similarly, clinical variant databases contain tens of thousands of pathogenic variants, many of which are observed in only a limited number of individuals [65]. This extensive mutational diversity poses a major challenge for mutation‐specific base editing therapies, as each variant may require individualized gRNA design, experimental validation, vector optimisation and regulatory evaluation. Such individualized development pipelines can substantially increase research and development costs and complicate large‐scale clinical implementation, particularly when compared with therapies targeting recurrent mutations or shared disease mechanisms. Given that the development of advanced genetic therapies often requires substantial financial investment, the economic feasibility of creating mutation‐specific editing strategies for rare variants remains uncertain. Recent research has demonstrated that ultra‐personalized genome‐editing strategies are technically achievable; however, the regulatory landscape and the logistics of large‐scale implementation present formidable hurdles to the widespread translation of these singular therapies [66, 67]. To resolve these challenges, a multifaceted approach is required that prioritizes the targeting of recurrent pathogenic variants alongside the development of mutation‐agnostic strategies, such as gene disruption or the modulation of regulatory elements. Furthermore, the successful translation of these technologies necessitates the establishment of adaptive regulatory frameworks specifically designed to support the unique requirements of individualized genomic therapies.

The capacity to perform accurate genome modifications also raises significant ethical questions. These include ensuring the safety of such edits, minimizing unintended genetic alterations, ensuring equitable access to potentially curative therapies, defining the line between therapeutic gene correction and genetic enhancement, and addressing the highly contentious issue of germline editing.

## 5 Recent Progress in Base Editing Technology

Base editing technology has undergone significant development following the introduction of early versions such as BE3 and ABE7.10. Protein engineering and directed evolution have been key drivers of this progress, aiming to overcome existing constraints and broaden the scope of these editing tools (Table 2).

### 5.1 Novel Editor Types

In addition to optimizing conventional CBEs and ABEs, recent discoveries have facilitated the development of entirely new classes of base editors with expanded capabilities (Table 2).

#### 5.1.1 Transversion Base Editors (CGBEs, AYBEs)

While standard base editors (CBEs and ABEs) perform transition mutations (swapping similar base types), creating transversion mutations (swapping purines for pyrimidines or vice versa) required new strategies. Cytosine‐to‐Guanine Base Editors (CGBEs), such as CGBE1 and Td‐CGBE, achieve C•G to G•C changes [37]. They typically combine a CBE structure (nCas9‐APOBEC1/TadA variant) with engineered DNA glycosylases (like modified UNG or TDG). These glycosylases remove the intermediate uracil base in a way that prompts DNA repair to insert guanine [47]. Td‐CGBE, a TadA‐8e‐derived cytosine‐to‐guanine base editor, achieves C•G‐to‐G•C conversion efficiencies of up to 72.8% and operates within a narrow editing window spanning nucleotides C5 to C6, providing high specificity for targeted genomic loci [46]. Its development expands the scope of base editing beyond transition mutations, enabling precise correction of previously inaccessible cytosine sites in mammalian genomes. Similarly, Adenine Transversion Base Editors (Adenine to Y (Pyrimidine) Base Editor (AYBEs)) use ABEs fused with engineered glycosylases to convert A•T pairs to T•A or C•G [68]. By fusing an evolved adenine deaminase (TadA‐8e) with a nickase Cas9 and N‐methylpurine DNA glycosylase (MPG) or alkyladenine DNA glycosylase (AAG), AYBEs enable precise A•T‐to‐C•G or A•T‐to‐T•A conversions within specific sequence contexts. The engineered mAAG (mouse AAG) excises the adenine‐derived intermediates, achieving A‐to‐C/T conversions of up to 73%, while maintaining a defined editing window and high locus specificity [69]. This development provides a powerful tool for correcting pathogenic point mutations that were previously inaccessible to conventional adenine base editors. Together, these transversion editors significantly broaden the types of point mutations that can be corrected.

#### 5.1.2 Dual Adenine and Cytosine Base Editors

Dual‐function base editors enable simultaneous A•T‐to‐G•C and C•G‐to‐T•A conversions within the same target DNA region. These tools are constructed by fusing both an adenine deaminase (like evolved TadA variants) and a cytidine deaminase (such as PmCDA1 or hA3A) to a nCas9 [70, 71]. Tools such as SPACE, A&C‐BEmax, STEMEs and pDuBE1 allow for making multiple different base changes simultaneously (like C•G to T•A and A•T to G•C) or generating intricate patterns of edits within targeted DNA segments [72]. A&C‐BEmax demonstrates robust cytosine editing with substantial adenine activity while reducing RNA off‐target effects, whereas SPACE, STEMEs and pDuBE1 facilitate programmable multi‐base substitutions, providing precise control over intricate DNA modifications.

#### 5.1.3 TadA‐Derived Cytosine Base Editors

The emergence of TadA‐derived CBEs (TadCBEs) represents a major advance in precision genome editing. Conventional CBEs such as BE3 and BE4 suffer from broad sequence context dependence, high frequencies of bystander edits and substantial Cas‐independent off‐target DNA and RNA deamination. To overcome these limitations, researchers repurposed TadA as a cytidine deaminase through directed evolution and structure‐guided mutagenesis. TadA variants, including the hyperactive TadA‐8e, were subjected to PACE and iterative site‐directed mutagenesis to shift substrate preference from adenine to cytosine, and the resulting TadCBEs were fused to nCas9 to generate programmable CBEs. Unlike APOBEC1, TadA's compact size (~166 amino acids) makes it favourable for delivery in therapeutic settings, including packaging into size‐constrained vectors such as Adeno‐associated viruses (AAVs) [73]. These engineered TadA mutants retain the structural fold of adenine deaminases but incorporate active site mutations that reconfigure base recognition and catalytic activity towards cytosine substrates. TadCBEs demonstrate comparable or higher editing efficiencies relative to APOBEC1‐CBEs, achieving 56% efficiency at target cytosines with clean editing windows typically spanning positions 4–8 relative to the PAM [73]. Importantly, transcriptome‐wide analyses have shown that TadCBEs exhibit negligible RNA off‐target editing, a significant improvement over APOBEC1‐CBEs. Moreover, their applicability extends to plant systems, where TadCBEa, TadCBEd and TadCBEd_V106W mediated efficient and precise C‐to‐T conversions with editing frequencies ranging from 4.5% to 55% [74].

Beyond single‐function CBEs, TadA engineering also enabled the development of dual editors such as TadDE, which simultaneously mediates C‐to‐T and A‐to‐G conversions within the same protospacer window, enabling multiplexed mutagenesis from a single editing event [74]. Similarly, CABE‐T (CABEs that use engineered variants of TadA) variants permit programmable cytosine and adenine editing with high precision, expanding both research and therapeutic applications, achieving ~53% average C‐to‐T editing efficiency with < 2% unintended A‐to‐G substitutions [75]. Together, these TadA‐derived CBEs combine high efficiency and precision with broad applicability across mammalian systems, animal models and crops, offering promising potential for therapeutic and agricultural genome engineering.

#### 5.1.4 Glycosylase‐Based Base Editors

Recent developments have introduced glycosylase‐based base editors (gGBEs, gTBEs and gCBEs), which utilize engineered DNA glycosylases fused with nCas9 to enable direct base editing that incorporates DNA glycosylase activity to expand editing outcomes beyond conventional deaminase‐based systems [76]. These editors offer precise control over base conversions, expanding the repertoire of editable sites in the genome.

The recently developed deaminase‐free guanine base editor, gGBE, was generated by fusing nCas9 with an engineered human MPG. The gGBE system uses the engineered MPG to specifically recognize and excise guanine, generating an apurinic/apyrimidinic (AP) site at the target locus. This abasic site is subsequently processed by endogenous DNA repair pathways, primarily TLS and BER, which insert a new base, typically T or C, at the damaged position. As a result, the original G•C base pair can be converted into C•G or T•A, enabling G‐to‐Y (Y = C or T) editing [77]. In mammalian cells, gGBE has achieved editing efficiencies of up to ~80% at certain genomic loci. Glycosylase‐Based Thymine Base Editors (gTBEs) employ a similar strategy, utilizing engineered thymine DNA glycosylase (TDG) variants fused to nCas9 to excise thymine bases, enabling T•A to G•C or T•A to C•G or T•A to A•T conversions [78]. In human cells, gTBE has demonstrated T‐to‐G editing efficiencies up to 48.7% and T‐to‐C editing up to 26.2%, with editing primarily occurring within positions 2–6 of the protospacer and maximal activity around position 3. Glycosylase‐Based Cytosine Base Editors (gCBEs) utilize engineered human UNG variants fused to nCas9 to generate abasic sites at targeted cytosines, thereby facilitating precise C•G to G•C transversion via the BER pathway. In mammalian cells gCBEs have achieved C‐to‐G editing efficiencies ranging from ~32% to ~78% across endogenous genomic loci [79]. Additionally, in rice, gCBEs utilizing either engineered hUNG or hUNG2 variants in conjunction with nCas9 have demonstrated C‐to‐G editing efficiencies ranging from 26.1% to 61.1%, with predominant C‐to‐G conversions reaching 58.3% and negligible off‐target activity [80]. These glycosylase‐based editors provide a versatile toolkit for precise base editing across all four nucleotides, offering advantages such as reduced off‐target effects and the ability to edit bases previously inaccessible to traditional deaminase‐based editors. Their development marks a significant advancement in the field of genome engineering, expanding the potential for therapeutic interventions targeting a broader range of genetic mutations.

#### 5.1.5 Organellar Base Editors

Genome editing within mitochondria and chloroplasts was historically difficult due to challenges in delivering CRISPR components, particularly gRNAs, into these organelles [81]. A major advancement came with CRISPR‐free base editors. These systems use alternative DNA‐targeting proteins, like TALEs or Zinc Finger Proteins, fused to deaminase enzymes, bypassing the need for gRNAs. For instance, DdCBEs utilize TALE proteins linked to split DddA cytidine deaminase to achieve C•G‐to‐T•A editing in mitochondrial DNA (mtDNA). Subsequently, mitochondrial ABEs (such as dTALED and mTALED) were created by integrating TadA variants into this TALE‐based framework to perform A•T‐to‐G•C edits in mtDNA [82]. These organellar editors open up new research and therapeutic possibilities for mitochondrial diseases and have also been adapted for editing plant chloroplast genomes.

#### 5.1.6 RNA Base Editors

RNA base editing allows for temporary and reversible alterations in gene expression because it targets RNA molecules, in contrast to genome editing techniques that permanently change the DNA [43]. Typically, these editors utilize an RNA‐targeting CRISPR system, like dCas13, fused to an RNA deaminase enzyme. Fusing adenosine deaminases (Adenosine Deaminase Acting on RNA (ADARs)) or engineered versions to dCas13 (e.g., dCas13b) enables A‐to‐I editing (interpreted as G), as demonstrated by the REPAIR (RNA Editing for Programmable A to I Replacement) system [43]. Versions like REPAIRv2 were developed to improve editing specificity and reduce off‐target effects. Alternatively, systems like RESCUE (RNA Editing for Specific C to U Exchange) use cytidine deaminases (e.g., APOBEC) to achieve C‐to‐U editing. The range of possible RNA modifications has recently expanded to include A‐to‐m6A and U‐to‐pseudouridine (Ψ), with newer systems like RtABE (RNA transformer Adenosine Base Editor) enhancing editing precision [83].

Although substantial progress has been made in developing diverse classes of base editors with expanded capabilities, the most mature and extensively optimized platforms remain CBEs and ABEs. These editors have undergone extensive engineering to improve their efficiency, target scope and specificity across multiple biological systems. In contrast, many of the recently developed base editing systems capable of generating transversion mutations or broader nucleotide substitutions are still in relatively early stages of development [76, 84]. While these emerging tools significantly expand the potential scope of genome editing, their efficiency, editing precision and levels of unintended byproducts remain important challenges that require further Optimisation before they can achieve the same level of reliability and therapeutic readiness as CBEs and ABEs.

### 5.2 Comparison With Prime Editing (PE)

Prime editing, another significant innovation in precision genome editing from David Liu's lab, operates differently from base editing (Figure 1E) [47]. Instead of direct base conversion, it uses a ‘search‐and‐replace’ mechanism [85]. Prime editors combine a nCas9 with a reverse transcriptase (RT) [4]. They are directed by a unique prime editing guide RNA (pegRNA) that both targets the editor to the correct DNA location and provides an RNA template encoding the desired genetic change, along with a primer binding site. After the Cas9 nicks the DNA, the exposed DNA end primes the RT, which then synthesizes an edited DNA sequence using the pegRNA's template. Finally, the cell's DNA repair processes integrate this new sequence into the genome [86].

Prime editing offers greater versatility than base editing, capable of performing all 12 types of base substitutions (both transitions and transversions) along with small insertions (up to ~44 bp) and deletions (up to ~80 bp) (Table 1) [26]. This broad scope theoretically enables prime editing to correct roughly 89% of identified disease‐causing human genetic variants. Additionally, it avoids the unintended ‘bystander’ mutations sometimes caused by base editors. However, prime editing efficiency can be highly variable depending on the target sequence, and its large, complex machinery (Cas9‐RT fusion and long pegRNA) presents delivery challenges [87]. In contrast, base editors are simpler in design and often achieve higher efficiency for the specific transition edits they are suited for, making them potentially preferable for correcting certain types of point mutations [88].

A clearer comparison between base editing and prime editing can be made across several key parameters (Table 1). Base editors generally provide higher editing efficiencies for transition mutations (C•G to T•A and A•T to G•C) and benefit from a simpler molecular architecture, which may facilitate delivery. In contrast, prime editing offers broader editing versatility by enabling all types of base substitutions as well as small insertions and deletions [86]. However, this increased versatility often comes with more variable editing efficiencies and greater delivery complexity due to the larger Cas9–RT fusion protein and the longer pegRNA required for editing.

The choice between base editing and prime editing depends on the specific application, balancing efficiency, precision and delivery constraints against the type of edit required and the genomic context. Base editing technology is characterized by its remarkable modularity and continuous development. By rationally designing or evolving components such as Cas9, deaminase, linkers and repair pathway elements (e.g., UGI), scientists have steadily improved base editor performance in terms of accuracy, targeting scope and customization. This adaptability has driven major innovations, including transversion and dual base editing, as well as extending the technology to edit DNA within organelles and modify RNA. Such progress demonstrates that base editing is a flexible platform, now capable of addressing mitochondrial mutations and exploring temporary RNA‐based therapies. Key frontiers for ongoing research include overcoming delivery hurdles for these advanced systems and gaining a deeper understanding of the biological consequences of RNA editing.

## 6 Applications and Clinical Translation

**FIGURE 3:**
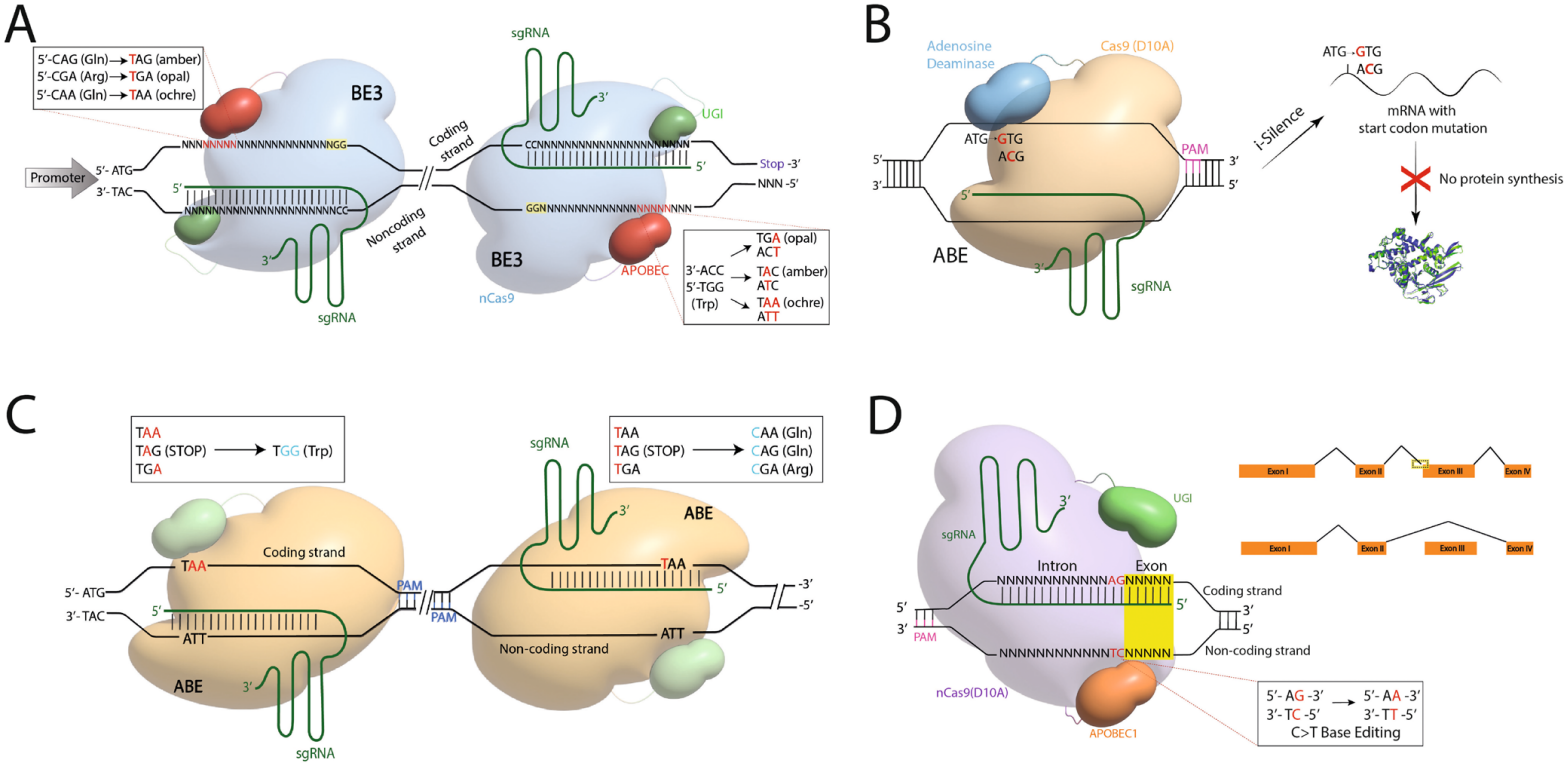
Diverse Applications of DNA Base Editing Technology [5]. DNA base editors enable precise nucleotide conversions (C•G to T•A by CBEs; A•T to G•C by ABEs) for various research and therapeutic purposes. (A) CRISPR‐STOP (or iSTOP) is a precise gene silencing technique that utilizes a base editor, specifically a CBE composed of a nCas9 guided by RNA and fused to a cytosine deaminase to permanently inactivate genes without cutting the DNA double helix. By targeting specific codons like CAA, CAG, or CGA within a gene, the CBE converts a cytosine (C) base into a thymine (T), thereby transforming the targeted codon into a premature stop codon (TAA, TAG or TGA). This early stop signal halts protein synthesis, effectively knocking out the gene's function. This diagram shows how the CRISPR‐STOP technique uses the BE3 base editor (APOBEC1‐nCas9‐UGI) to convert specific codons into STOP codons, thereby inactivating genes [89]. The upper illustration details the editing of Arginine (Arg) and Glutamine (Gln) codons on the coding strand. The lower illustration shows the editing of a Tryptophan (Trp) codon via targeting the non‐coding strand. In both cases, BE3 introduces the STOP signal. UGI stands for uracil glycosylase inhibitor. (B) I‐Silence is an innovative gene silencing approach that employs ABEs to precisely alter the start codon ATG into non‐functional sequences such as GTG or ACG, thereby preventing the initiation of protein translation [90]. Unlike conventional gene knockout methods that depend on double‐stranded DNA breaks, i‐Silence provides a safer and more precise alternative by avoiding these breaks and minimizing the potential for off‐target mutations. (C) CRISPR‐Pass is a base editing technique designed to rescue genes affected by premature stop codons (nonsense mutations) by using ABEs to convert these stop signals into amino acid‐encoding codons. This targeted change allows the ribosome to bypass the erroneous stop signal and continue translating the full‐length protein, effectively restoring gene function. Unlike traditional gene editing methods that rely on DSBs, CRISPR‐Pass offers a precise and less invasive approach, making it especially promising for treating genetic disorders caused by nonsense mutations. A schematic illustrating ABE‐mediated CRISPR‐Pass is presented [91]. Targetable adenines reside on either the coding or noncoding strand, depending on the orientation of the PAM sequence. The upper boxes illustrate all possible premature termination codons (PTCs) that can be targeted: TAA, TAG and TGA for both coding strand and noncoding strand targeting. (D) CRISPR‐SKIP is a base editing approach that modifies splice sites to cause targeted exon skipping during RNA processing. By using cytosine or adenine base editors to disrupt key nucleotides at splice donor or acceptor sites, it prevents the normal inclusion of specific exons in the final mRNA. Without introducing DSBs, this method can restore proper gene function in certain genetic diseases, reduce gene activity, or facilitate the study of splicing regulation. CRISPR‐SKIP targeting strategy [92]. The splice acceptor site contains a conserved sequence, with a highly conserved guanosine. It is hypothesized that base editing this guanosine can trigger exon skipping. When a suitable PAM sequence is present, base editors can deaminate a cytidine on the antisense DNA strand, which pairs with the conserved guanosine on the sense strand. This modification disrupts the splice acceptor site, thereby promoting exon skipping during RNA splicing.

Base editors constitute a substantial leap forward in gene editing, offering the ability to introduce precise point mutations without inducing potentially harmful DSBs. This precision makes them invaluable tools across diverse fields such as molecular medicine, functional genomics and agriculture (Figure 3) [5]. A major application lies in human therapeutics, where base editors show great potential in directly correcting pathogenic point mutations responsible for numerous genetic disorders. Their ability to precisely target and convert single nucleotides offers a direct route to potentially cure monogenic diseases. Specifically, ABEs can correct nonsense mutations (premature termination codons—PTCs like TAA, TAG, TGA) by converting the adenine (A) to guanine (G), changing the stop codon (CRISPR‐Pass, Figure 3C) into one encoding an amino acid (e.g., TAG to TGG (Trp)) [91]. While this might not always restore the original wild‐type amino acid, it allows for the generation of full‐length protein molecules, potentially rescuing function.

Conversely, base editors are instrumental in creating accurate genetic models of human diseases. By introducing specific disease‐causing SNVs into cell lines or animal models, researchers can rigorously investigate disease mechanisms and evaluate potential therapies. For instance, cancer‐associated mutations in genes like *TP53*, *CTNNB1* (β‐catenin) and the *TERT* promoter have been successfully introduced into human induced pluripotent stem cells (iPSCs) and mouse models to study tumour initiation and drug responses in cancers like breast and liver cancer [93]. Furthermore, sophisticated in vivo models, such as inducible base editing (iBE) mouse systems, allow for temporally controlled introduction of SNVs in specific tissues using doxycycline‐dependent activation of a CBE. This enables the rapid generation of precise preclinical cancer models in organs like the intestine, lung and pancreas, facilitating studies on tumour progression and the functional impact of specific cancer‐associated SNVs [94].

Base editing also provides precise methods for gene knockout or disruption. CBEs can be targeted to codons like CAA (Gln), CAG (Gln), CGA (Arg) or the sequence complementary to TGG (Trp). Editing the cytosine (C) in these codons converts them into premature stop codons (TAA, TAG or TGA), leading to truncated mRNA or non‐functional proteins, effectively knocking out gene function. Systems like CRISPR‐STOP (or iSTOP) leverage this principle for efficient gene disruption (Figure 3A) [89]. Similarly, ABEs can disrupt gene function by targeting the adenine (A) in the canonical ATG start codon, converting it to GTG (Val) or otherwise disrupting the Kozak sequence context, thereby inhibiting translation initiation (i‐Silence‐ Figure 3B) [90]. Beyond coding regions, base editing can modulate mRNA splicing. CBEs can target the cytosine on the non‐coding strand corresponding to a critical guanine (G) in splice acceptor sites. The resulting C‐to‐T edit on the DNA leads to a G‐to‐A mutation in the RNA splice site sequence, often causing aberrant splicing events like exon skipping or intron retention, altering the final protein product (CRISPR‐SKIP, Figure 3D) [92].

Base editors are powerful tools for functional genomics and high‐throughput screening. They enable saturation mutagenesis, creating comprehensive libraries of variants to map protein structure–function relationships or evolve proteins with novel capabilities [15]. Large‐scale screens using base editing can systematically assess the functional consequences of individual nucleotide changes, identifying mutations conferring drug resistance or mapping crucial protein domains. For example, CRISPR base editing mutagenesis screens in cancer cell lines have been used to probe resistance mechanisms to various oncology drugs by generating tens of thousands of specific variants across key cancer genes [95].

In agriculture, base editing is being used to enhance crop characteristics through precise single‐base substitutions. This technology has been applied to introduce beneficial traits such as herbicide resistance (e.g., by targeting *OsALS1* in rice), improved grain quality (e.g., modifying *Wx* alleles in rice) and increased nutritional value in crops including tomato, maize, wheat and rice [14, 37]. In summary, the ability of base editors to precisely alter single nucleotides without causing DSBs underpins their broad utility, from developing new human gene therapies and modelling diseases with high fidelity to advancing functional genomics screens and improving agricultural traits.

### 6.1 In Vivo Delivery Strategies and Challenges

**FIGURE 4:**
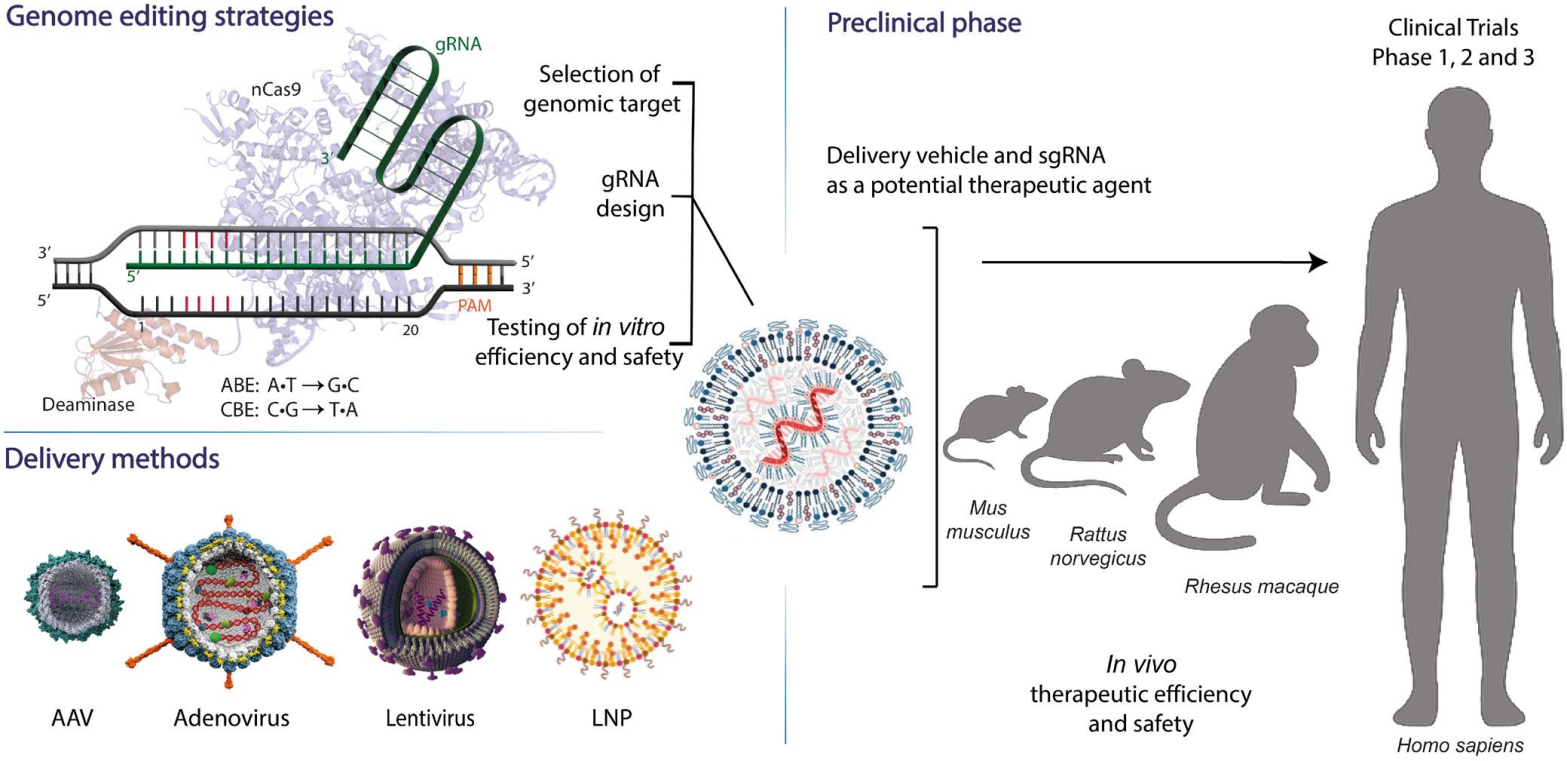
Overview of the Development Pipeline for Base Editing Therapies [61]. This schematic illustrates the key stages in translating base editing technology into clinical applications. The pipeline initiates with target identification and rational design of the base editor protein and guide RNA, coupled with the parallel development of effective delivery methods (e.g., viral vectors, LNPs). Initial preclinical validation is conducted in vitro using relevant cell models to assess on‐target editing efficiency and off‐target specificity. Subsequent in vivo preclinical testing in animal models evaluates therapeutic efficacy, confirms delivery to target tissues and assesses overall safety and biodistribution. Data from preclinical studies drive iterative Optimisation of the editor components and delivery system. Following comprehensive evaluation and demonstration of potential, regulatory submissions are prepared for approval to initiate clinical trials in humans, which proceed through phases (Phase 1–3) to establish safety and efficacy. Successful clinical validation can lead to regulatory approval for therapeutic use in patients.

Delivering the base editing tools the Cas9‐deaminase fusion protein or its mRNA, along with the gRNA safely, effectively and precisely to target tissues in vivo remains a primary obstacle for the clinical use of base editing, especially for treating systemic diseases (Figure 4) [3]. This difficulty is partly related to the structural complexity and relatively large size of base editor systems, which can complicate efficient packaging and delivery to target tissues. As a result, achieving efficient, safe and tissue‐specific in vivo delivery remains a major challenge for the therapeutic application of base editing technologies.

#### 6.1.1 Viral Vectors

Viral vectors utilize viruses' inherent capacity to infect cells to deliver genetic material. However, different types of viral vectors offer distinct benefits and drawbacks. AAVs are a top choice for in vivo gene delivery, valued for their low immunogenicity, ability to infect non‐dividing cells and predictable patterns of tissue targeting [26]. However, their significant limitation is a small packaging capacity (around 4.7 kb) [96], which struggles to accommodate large base editor systems based on SpCas9 (over 4 kb). Because of this size constraint, base editor constructs often approach or exceed the packaging capacity of single AAV vectors. To circumvent this size limit, dual‐AAV strategies are commonly used, splitting the base editor components across two vectors [62]. Each fragment is fused to a part of a split intein; upon co‐delivery into a cell, these inteins mediate protein splicing to reassemble the complete, functional editor protein. Despite these advances, dual‐vector systems require efficient co‐transduction and intracellular reconstitution of the editor, which may reduce overall editing efficiency and complicate therapeutic implementation. This dual‐vector approach has successfully enabled in vivo base editing in various tissues (including liver, muscle, brain, eye, inner ear) in mouse models of diseases like progeria, SMA, DMD, ALS, LCA, hereditary deafness and PKU [27]. Despite this success, AAV delivery often results in prolonged expression of the base editor from persistent episomal DNA. This raises concerns about potential cumulative off‐target editing and the risk of immune responses against the Cas9 protein or AAV capsid, which could eliminate edited cells or preclude repeat administrations [61]. Lentiviruses (LVs) provide a substantial cargo capacity (~10 kb), suitable for delivering intact base editors and multiple gRNAs [62]. However, their integration into the host genome poses significant safety risks like insertional mutagenesis and genotoxicity, currently hindering their in vivo clinical application [97]. LVs are still valuable preclinical tools, though, as shown by their use in correcting the *Rpe65* gene with an ABE in a mouse model of LCA [27]. Adenoviruses (AdVs) offer a very large cargo capacity, up to 36 kb, making them capable of carrying sizable or intricate base editor systems. However, their tendency to provoke strong immune responses (high immunogenicity) limits their usefulness for applications requiring systemic delivery [98]. Despite this limitation, AdVs have been utilized in some specific preclinical studies exploring base editing technique [27, 99].

#### 6.1.2 Non‐Viral Delivery Systems

LNPs are a leading non‐viral system for delivering nucleic acids, including the components needed for base editing [100]. A typical LNP formulation includes four key elements: an ionizable lipid essential for releasing the cargo from cellular compartments (endosomal escape), helper lipids, cholesterol and a PEG‐lipid to improve stability and reduce detection by the immune system [63]. LNPs offer significant advantages compared to viral vectors [101]. These include generally lower immunogenicity, the flexibility to deliver different types of cargo like mRNA or pre‐assembled RNP complexes (Cas protein plus gRNA), suitability for repeated administrations and the promotion of transient base editor expression [36]. Delivering the editor as an RNP complex, in particular, results in short‐lived activity, which minimizes the risk of off‐target edits and avoids concerns related to integrating genetic material into the host genome. Nevertheless, non‐viral delivery systems still face important challenges related to delivery efficiency, tissue specificity and long‐term safety, which remain areas of active investigation for therapeutic base editing applications.

LNPs efficiently deliver their contents into the cytoplasm of target cells [39]. They possess a natural tendency to accumulate in the liver, largely due to interactions involving apolipoprotein E (ApoE) binding and uptake using the low‐density lipoprotein receptor (LDLR) pathway. This inherent liver tropism makes them particularly effective for hepatic gene editing, a property leveraged by companies like Verve and Beam in programs targeting genes such as *PCSK9*, *ANGPTL3*, *G6PC* and *SERPINA1*. To enable targeting beyond the liver, current research focuses on engineering LNPs. This includes developing tailored lipid compositions (like SORT LNPs designed for lung and spleen delivery) and adding specific molecules (such as antibodies) to the LNP surface to direct them towards particular cell types, like T cells [102]. Beyond established viral and LNP systems, researchers are actively investigating a range of other strategies to deliver base editing tools, aiming to expand the options for therapeutic application [99].

Virus‐Like Particles (VLPs) are non‐infectious particles derived from viral scaffolds (e.g., retroviruses) engineered to package and deliver protein or RNP cargos, including base editors. VLPs offer the potential for transient delivery of the pre‐assembled base editor RNP complex, minimizing off‐target risks associated with prolonged expression from DNA or mRNA templates [103]. Consequently, directed evolution strategies are being used to improve VLP packaging and delivery efficiency. Furthermore, ENVLPE (Engineered Nucleocytosolic Vehicles for Loading of Programmable Editors) is a cutting‐edge gene‐editing delivery platform that employs non‐infectious VLPs to efficiently deliver gene‐editing tools such as CRISPR‐Cas9, base editors and prime editors into target cells. This advanced VLP‐based system offers programmable, non‐viral delivery and significantly enhances editing efficiency in challenging cell types and in vivo models [104].

Extracellular Vesicles (EVs) are naturally formed nanoscale particles that facilitate communication between cells. Their biocompatibility, low immunogenicity and capacity to cross biological barriers make them attractive candidates for non‐invasive delivery of base editing cargo [105]. Delivering the base editor as an RNP offers the most transient exposure, further enhancing safety by limiting the time window for off‐target activity [106]. Physical methods, such as electroporation (frequently used in ex vivo cell therapy) or nanoneedles, enable the direct introduction of pre‐assembled base editor RNP complexes into cells [107]. This offers highly controlled delivery and transient editor activity. However, difficulties in achieving broad tissue access and efficiency generally limit these techniques to modifying cells outside the body (ex vivo) or for localized treatments in vivo. While electroporation is commonly used for RNP delivery ex vivo, in vivo delivery typically requires packaging within vehicles like LNPs or VLPs. Recent studies suggest packaged RNP delivery (e.g., via enveloped delivery vehicles, EDVs) can be significantly more efficient than electroporation at lower doses [108].

### 6.2 Therapeutic Applications: From Preclinical Validation to Clinical Translation

Base editing technology holds transformative potential for genomic medicine, offering a precise way to correct the single‐letter DNA errors or point mutations, that underlie many inherited human diseases. Before this powerful tool can be used for patients, it must first be strictly validated through preclinical research [27]. These essential studies, often conducted in cell lines and relevant animal models mirroring human conditions, serve as the crucial proving ground for therapeutic base editing strategies. They provide the necessary in vivo proof‐of‐concept, demonstrating not only that the base editor can successfully reach target tissues and correct the specific genetic defect, but also that this correction leads to a tangible improvement in disease symptoms or biomarkers. Therefore, the success observed in many preclinical studies where base editing improved model conditions such as progeria, metabolic disorders and sickle cell disease is vitally important, as it builds the core efficacy and safety evidence needed to justify progressing these therapies to human clinical trials. Building on extensive preclinical validation, base editing technology is advancing into human clinical trials, marking a significant milestone in the clinical translation of precision genome editing (Table 3). These initial human studies are primarily focused on monogenic diseases, utilizing either ex vivo approaches where patient cells are edited outside the body or in vivo strategies often targeting the liver [109].

**TABLE 3:** Overview of Clinical Trials Employing Base Editing Technologies (ABE/CBE) as of March 2026.

NCT ID	Therapy name/identifier	Target indication(s)	Base editor type	Editing strategy	Delivery method	Target cell/tissue	Sponsor(s)	Phase	Recruitment status (as of date/source)
NCT05398029	VERVE‐101	HeFH, ASCVD	ABE (ABE8.8 m)	Gene inactivation (*PCSK9*)	In vivo, LNP	Hepatocytes	Verve Therapeutics Inc	Phase 1b	Completed (May 2025)
NCT06164730	VERVE‐102	HeFH, Premature CAD	ABE	Gene inactivation (*PCSK9*)	In vivo, GalNAc‐LNP	Hepatocytes	Verve Therapeutics Inc	Phase 1b	Recruiting (Feb 2026)
NCT06451770	VERVE‐201	Refractory Hypercholesterolemia (RH), HoFH	ABE	Gene inactivation (*ANGPTL3*)	In vivo, GalNAc‐LNP	Hepatocytes	Verve Therapeutics Inc	Phase 1b	Recruiting (Nov 2025)
NCT05456880	BEAM‐101	Sickle Cell Disease (SCD) (Severe VOCs)	ABE	Promoter editing (HBG1/2, mimic HPFH)	Ex vivo, Electroporation	HSPCs	Beam Therapeutics Inc	Phase 1/2	Active, Not recruiting (Dec 2025)
NCT06024876	CS‐101	Transfusion‐Dependent β‐Thalassaemia (TDT)	Transformer Base Editor (tBE/CBE)	Disrupt BCL11A binding site in HBG promoter	Ex vivo, Electroporation	HSPCs	Correct Sequence Therapeutics (China)	Phase 1	Completed (Feb 2026)
NCT05885464	BEAM‐201	T‐ALL/T‐LL (Relapsed/Refractory, CD7+)	CBE	Multiplex knockout (CD7, TRAC, PDCD1, CD52)	Ex vivo, Electroporation	Allogeneic T cells	Beam Therapeutics Inc	Phase 1/2	Active, Not recruiting (Dec 2025)
NCT06735755	BEAM‐301	Glycogen Storage Disease Type Ia (GSDIa)	ABE	Gene correction (G6PC R83C mutation)	In vivo, LNP	Hepatocytes	Beam Therapeutics Inc	Phase 1/2	Recruiting (Dec 2025)
NCT06389877	BEAM‐302	Alpha‐1 Antitrypsin Deficiency (AATD) (PiZZ, Lung/Liver)	ABE	Gene correction (*SERPINA1 PiZ* allele)	In vivo, LNP	Hepatocytes	Beam Therapeutics Inc	Phase 1/2	Recruiting (July 2025)
NCT05397184	TvT CAR7 (BE CAR‐7)	T‐ALL (Paediatric, R/R, CD7+)	CBE	Multiplex knockout targeting *TRAC, CD52, CD7*	Ex vivo, Electroporation	Allogeneic T cells	GOSH/UCL/MRC (UK)	Phase 1	Unknown Status (July 2023)
NCT06325709	NIAID X‐CGD Therapy	X‐linked Chronic Granulomatous Disease (X‐CGD)	ABE (ABE8e‐SpRY)	Gene correction (CYBB missense mutations, e.g., c.676C>T)	Ex vivo (Electroporation likely)	HSPCs	National Institute of Allergy and Infectious Diseases (NIAID)	Phase 1/2	Recruiting (March 2026)
NCT07176923	CS‐121	Familial Chylomicronemia Syndrome (FCS)	tBE/CBE	Gene inactivation (*APOC3*)	In vivo, LNP	Hepatocytes	Correct Sequence Therapeutics (China)	Early Phase 1	Recruiting (Feb 2026)
NCT06565026	CS‐206	SCD	tBE/CBE	Disrupt *BCL11A* binding site in HBG promoter	Ex vivo, Electroporation	HSPCs	Correct Sequence Therapeutics (China)	Early Phase 1	Recruiting (Feb 2026)
NCT06392724	GEN6050X	Duchenne Muscular Dystrophy (DMD)	CBE	Exon 50 skipping in *DMD* gene	In vivo, Dual ss.AAV9	Skeletal and Cardiac Muscles	GenAssist Therapeutics (China)	Early Phase 1	Active, not recruiting (July 2025)
NCT06860672	Dual Vector BE for CHD3‐R1025W	Snijders Blok‐Campeau Syndrome	ABE	Gene correction (*CHD3* c.3073C>T; p.R1025W)	In vivo, Dual AAV	Central Nervous System (CNS)	Yongguo Yu/Xinhua Hospital	Early Phase 1	Recruiting (March 2025)
NCT07353398	ART002g1	HeFH	ABE	Gene inactivation (*PCSK9*)	In vivo, LNP	Hepatocytes	AccurEdit Therapeutics (China)	Early Phase 1	Not yet recruiting (Feb 2026)
NCT06458010	YOLT‐101	HeFH	ABE	Gene inactivation (*PCSK9*)	In vivo, LNP	Hepatocytes	YolTech Therapeutics (China)	Early Phase 1	Recruiting (April 2025)
NCT06959771	NIAID *CD40L* Therapy	X‐linked Hyper IgM (HIGM)	ABE (ABE8e)	Gene correction (*CD40L* c.658C>T; p.Q220X)	Ex vivo, Electroporation	HSPCs	NIAID	Phase 1/2	Recruiting (Jan 2026)

*Note:* The report summarizes essential trial characteristics, including the ClinicalTrials.gov Identifier (NCT ID), the therapeutic candidate's name, targeted disease indication(s), the specific type of base editor and editing strategy employed, the delivery method (in vivo or ex vivo), the sponsoring organization(s), the trial phase and the latest known recruitment status.

Abbreviations: ABE, Adenine Base Editor; ASCVD, Atherosclerotic Cardiovascular Disease; CAD, Coronary Artery Disease; CAR‐T, Chimeric Antigen Receptor T cell; CBE, Cytosine Base Editor; GOSH, Great Ormond Street Hospital; HeFH, Heterozygous Familial Hypercholesterolemia; HoFH, Homozygous Familial Hypercholesterolemia; HPFH, Hereditary Persistence of Fetal Haemoglobin; HSPC, Haematopoietic Stem and Progenitor Cell; KO, Knockout; LDL‐C, Low‐Density Lipoprotein Cholesterol; LNP, Lipid Nanoparticle; MRC, Medical Research Council; NCT, National Clinical Trial; NIAID, National Institute of Allergy and Infectious Diseases; NZ, New Zealand; PCSK9, Proprotein Convertase Subtilisin/Kexin type 9; R/R, Relapsed/Refractory; SCD, Sickle Cell Disease; T‐ALL, T‐cell Acute Lymphoblastic Leukaemia; T‐LL, T‐cell Lymphoblastic Lymphoma; TCR, T‐Cell Receptor; TRAC, T‐Cell Receptor Alpha Constant; UCL, University College London; UK, United Kingdom; US, United States; VOC, Vaso‐Occlusive Crisis.

#### 6.2.1 Experimental and Non‐Clinical Delivery Approaches

Certain delivery strategies, such as hydrodynamic injection, are primarily used in preclinical settings to establish proof‐of‐concept for in vivo genome editing but are not directly translatable to clinical applications. Hydrodynamic tail‐vein injections have been widely used in preclinical studies as an experimental delivery strategy for liver‐directed genome editing.

A representative example is hereditary tyrosinemia type I (HTI), a recessively inherited metabolic disorder affecting tyrosine metabolism, caused by a deficiency of the enzyme fumarylacetoacetate hydrolase (FAH) due to mutations in the *FAH* gene [110, 111]. This leads to the buildup of harmful metabolites (fumarylacetoacetate, succinylacetone) causing severe liver damage, renal tubulopathy and increased risk of hepatocellular carcinoma. Mouse models of HTI, often carrying a G•C to A•T point mutation in the last nucleotide of *Fah* exon 8 causing splicing defects, are widely used for testing liver‐directed therapies.

The potential for in vivo correction of this *Fah* splice‐site mutation was explored in adult HTI mice employing the ABE6.3 base editor [112]. The editor, encoded on plasmid DNA along with the sgRNA, was delivered systemically via hydrodynamic tail‐vein injection, a method that efficiently targets hepatocytes. Deep sequencing revealed an average A•T to G•C correction efficiency of ~9.5% in liver DNA, with very low indel formation (0.05%). Despite this relatively modest initial editing rate, the treatment led to significant phenotypic rescue. Following the withdrawal of the protective drug nitisinone, ABE‐treated mice maintained body weight, whereas control mice experienced rapid weight loss. Immunohistochemistry showed numerous patches of FAH‐positive hepatocytes throughout the liver parenchyma of treated mice. This occurs because corrected hepatocytes gain a strong selective survival and proliferative advantage in the diseased liver environment, allowing them to clonally expand and repopulate the organ over time. RT‐PCR confirmed partial restoration of correctly spliced *Fah* mRNA, and some treated mice survived long‐term without nitisinone. Another study demonstrated successful correction and therapeutic benefit using ex vivo ABE treatment of chemically derived hepatic progenitors followed by transplantation into HTI mice [113].

The HTI studies exemplify how even partial in vivo correction can lead to profound therapeutic benefit in the context of liver diseases that favour the proliferation of corrected cells. This suggests that achieving extremely high editing efficiencies may not be necessary for all indications, potentially lowering the threshold for clinical success.

#### 6.2.2 Controlled Precision: Ex Vivo Engineering and Hematologic Trade‐Offs

Ex vivo editing of patient‐derived or donor‐derived cells represents one of the most clinically advanced delivery paradigms for base editing therapies. In this approach, target cells are isolated, genetically modified outside the body under controlled conditions and subsequently reinfused into the patient. This strategy allows precise control over editing efficiency, quality control and safety assessment prior to transplantation. Ex vivo approaches are particularly suitable for haematological diseases, where haematopoietic stem and progenitor cells (HSPCs) can be readily isolated, edited and reintroduced following conditioning regimens.

One of the most extensively studied applications of ex vivo base editing is in sickle cell disease (SCD). SCD is an inherited blood condition caused by an A•T to T•A transversion (GAG to GTG) in the β‐globin gene (*HBB*), causing glutamic acid to be replaced by valine at position six of the β‐globin protein (E6V). This mutation produces sickle haemoglobin (HbS), which polymerizes under hypoxic conditions, deforming red blood cells (RBCs) into a sickle shape. These deformed cells lead to vaso‐occlusion, chronic hemolytic anaemia and progressive organ damage. Base editing presents two main therapeutic strategies for SCD: direct correction of the pathogenic mutation or reactivation of fetal haemoglobin (HbF) expression.

One strategy involves employing an ABE to change a pathogenic T•A base pair into a C•G pair by targeting the adenine specifically on the template strand [104]. Although this does not restore the wild‐type sequence (a T>A transversion inaccessible to standard ABEs), it introduces a benign variant known as haemoglobin Makassar. Using an ex vivo approach, haematopoietic stem cells (HSCs) isolated from SCD patients or an SCD mouse model were treated with the ABE, delivered as mRNA or RNP complexes, prior to transplantation into recipient mice. Editing efficiencies reached up to 80% in patient‐derived HSCs. Post‐transplantation, edited cells demonstrated successful engraftment with stable editing levels (~68% in mouse HSCs at 16 weeks). Phenotypic analysis revealed normalization of hematologic parameters, reduced RBC sickling and resolution of hallmark SCD symptoms such as anaemia and splenomegaly. Notably, correcting more than 20% of alleles was sufficient to confer therapeutic benefit in the mouse model.

An alternative therapeutic strategy involves reactivating fetal haemoglobin (HbF, α₂γ₂), which interferes with HbS polymerization and mitigates SCD symptoms. This is achieved by disrupting repressor binding motifs such as those for *GATA1* within the erythroid‐specific enhancer of the *BCL11A* (B cell lymphoma/leukaemia 11A gene) gene, a key modulator of the fetal‐to‐adult haemoglobin switch. Recent studies have utilized base editors (CBEs, ABEs, or dual editors) delivered ex vivo via electroporation of RNPs or RNA transfection to introduce precise nucleotide substitutions within the +58 kb or +55 kb enhancer regions in SCD patient‐derived HSPCs [39]. These approaches achieved efficient single‐site or multiplex editing (up to ~70% for multiplexed edits) with minimal DSB formation or large genomic rearrangement issues commonly associated with Cas9 nuclease‐based editing [114]. Base editing resulted in robust HbF reactivation, with HbF protein levels reaching ~29% in vitro, a level considered therapeutically effective. Edited cells displayed reduced sickling under hypoxia and retained high engraftment capacity in xenotransplantation models, with sustained HbF expression in vivo. Another strategy involves editing the promoter regions of the *HBG1* and *HBG2* genes to prevent repressor proteins from binding. This approach is designed to mimic naturally occurring HPFH mutations that lead to increased HbF levels [115].

This ex vivo editing paradigm has already progressed to clinical evaluation. BEAM‐101 is an investigational autologous HSC‐based therapy developed by Beam Therapeutics for the treatment of severe SCD. This therapy involves editing a patient's own HSCs ex vivo with an ABE. The edit introduces beneficial mutations, similar to those found in HPFH, into the *HBG1/2* promoter to increase protective HbF levels [116]. The ABE enzyme and the specific sgRNA targeting the *HBG1/2* promoters are delivered into the HSPCs using electroporation. Although the specific ABE variant used in the final BEAM‐101 product is proprietary, Beam Therapeutics has disclosed the development of advanced ABE versions, such as the ABE8 family, which demonstrate improved editing efficiency and specificity over earlier versions. The goal of BEAM‐101 editing is to trigger a strong, lasting and widespread reactivation of HbF production, leading to significant levels of HbF in the patient's mature red blood cells. Maintaining sufficiently high levels of HbF is predicted to impede the polymerization of HbS. This, in turn, should reduce red blood cell sickling, lessen chronic red blood cell destruction (hemolysis), prevent blood vessel blockages (vaso‐occlusion) and ultimately relieve the symptoms and complications of SCD.

The BEACON Phase 1/2 clinical trial (NCT05456880) is an open‐label, single‐arm, multicenter study designed to assess the safety and efficacy of BEAM‐101 in patients with SCD suffering from severe vaso‐occlusive crises (VOC) [117]. The ongoing BEACON Phase 1/2 trial has enrolled over 35 patients (8 dosed by late 2024) and recently expanded to adolescents [118]. Initial results presented in late 2024 indicated successful engraftment, increased HbF production, and improved red blood cell function. Updated results are anticipated by mid‐2025 [95]. These updates will provide further insights into the durability of HbF induction, the impact on clinical outcomes like VOCs and transfusions and the continued safety profile of BEAM‐101. While BEAM‐101's primary indication is SCD, its HbF induction strategy is directly applicable to β‐thalassaemia, as both SCD and β‐thalassaemia are hemoglobinopathies, and elevating HbF levels can ameliorate symptoms in both conditions [119].

A similar ex vivo genome editing strategy is also being applied to β‐thalassaemia, a group of inherited blood disorders manifested by absent or decreased production of beta‐globin chains in haemoglobin, resulting in ineffective erythropoiesis and anaemia of varying severity. Management of transfusion‐dependent β‐thalassaemia (TDT) usually requires lifelong blood transfusions and iron chelation therapy, both of which impose substantial burdens and pose risks of long‐term complications. Allogeneic HSCT provides a potential cure but is constrained by limited donor availability and risks related to immune rejection. Recent progress in genome editing, especially CRISPR‐based technologies, holds promise for transformative, one‐time curative treatments through modification of a patient's own HSCs [120]. Within these technologies, base editing has stood out as an especially promising method [121].

The CS‐101 clinical trial (NCT06024876) is a Phase 1, open‐label study assessing the safety and effectiveness of an autologous CD34^+^ haematopoietic stem cell therapy genetically engineered using cytosine base editing. Utilizing a novel transformer Base Editor (tBE), the protocol introduces precise single‐nucleotide edits in the *HBG1/2* promoters to disrupt the *BCL11A* binding motif, thereby derepressing γ‐globin expression and elevating HbF levels in patients with TDT. Interim results reported in 2024 for the first six treated subjects demonstrated robust haematopoietic engraftment, transfusion independence, a marked increase in total haemoglobin, and HbF predominance, without treatment‐related serious adverse events [122]. The trial represents the first clinical application of base editing in β‐hemoglobinopathies and could provide a durable, potentially curative therapy for TDT.

The ex vivo editing framework is also being expanded beyond hemoglobinopathies to engineered cell therapies in oncology. T‐cell acute lymphoblastic leukaemia (T‐ALL) and T‐cell lymphoblastic lymphoma (T‐LL) are aggressive haematological cancers that arise from T‐lymphocyte precursors. Despite significant advances in frontline therapies leading to improved survival rates, outcomes for patients with relapsed or refractory (R/R) T‐ALL/T‐LL remain poor [123]. Although chimeric antigen receptor (CAR) T‐cell therapy has revolutionized the treatment of B‐cell malignancies, applying it to T‐cell cancers presents unique challenges. These hurdles include the difficulty of selecting target antigens found on malignant T‐cells but largely absent from normal immune cells, as well as the problem of CAR‐T cell fratricide, where the therapeutic cells eliminate each other due to mutual expression of the target antigen. Moreover, minimizing the risk of Graft‐versus‐Host Disease (GvHD) remains a key concern when employing allogeneic (donor‐derived) T cells. Allogeneic CAR‐T therapies offer the potential for an ‘off‐the‐shelf’ product, addressing the manufacturing limitations associated with autologous therapies. However, achieving this potential requires sophisticated genetic engineering to ensure both safety and efficacy. BEAM‐201, developed by Beam Therapeutics, is a next‐generation allogeneic CAR‐T therapy that utilizes CBE to disrupt multiple genes in donor‐derived T cells, addressing major challenges in treating R/R T‐ALL and T‐LL. Through precise CBE‐mediated editing of four target genes, it aims to generate a potent, safe and universally applicable “off‐the‐shelf” product targeting CD7‐positive T‐ALL/T‐LL [124]. The multiplex editing strategy enhances both the safety and efficacy of the therapy by targeting key genes in the CAR‐T cells. The *CD7* knockout prevents fratricide by removing CD7 expression on the CAR‐T cells, allowing them to effectively target CD7‐positive T‐ALL/T‐LL cells without harming each other. The *TRAC* (T Cell Receptor Alpha Constant) knockout reduces the risk of GvHD by disrupting the TCR complex, preventing donor T cells from attacking the recipient's tissues. The *CD52* knockout resists lymphodepletion therapy, ensuring that BEAM‐201 cells are not depleted by treatments like alemtuzumab. Lastly, knocking out *PDCD1* reduces T cell exhaustion by removing the PD‐1 receptor, thereby blocking inhibitory signals and improving CAR‐T cell persistence and activity within the tumour microenvironment. Together, these edits optimize BEAM‐201's safety, persistence, and therapeutic potential in treating CD7‐positive T‐ALL/T‐LL. BEAM‐201 is currently undergoing evaluation in a Phase 1/2, multicenter, open‐label clinical trial (NCT05885464) targeting patients with R/R T‐ALL or T‐LL. The trial includes dose‐exploration and dose‐expansion cohorts for adults, as well as a planned cohort for paediatric patients. A Phase 1/2 clinical trial is actively enrolling and treating patients, with early data shared in late 2024 [125].

BEAM‐201 demonstrates the promise of base editing technology in developing complex, multiplex‐engineered cell therapies. By precisely introducing four distinct gene edits, BEAM‐201 aims to create a more effective and potentially safer allogeneic CAR‐T therapy for T‐ALL/T‐LL, addressing the significant unmet need in relapsed/refractory cases. Early data from the ongoing Phase 1/2 trial show a manageable safety profile and promising signs of efficacy, supporting further investigation. Additional clinical evaluation is essential to fully determine BEAM‐201's safety, efficacy and durability and to define the future role of multiplex base editing in CAR‐T and other cell‐based therapies.

Together, these examples illustrate how ex vivo base editing approaches enable precise genome engineering in clinically accessible cell types such as haematopoietic stem cells and T cells. By allowing editing, quality control and expansion outside the body, this delivery paradigm provides a controlled and scalable pathway towards therapeutic genome editing.

#### 6.2.3 Overcoming Packaging Constraints: Single‐ and Dual‐AAV Delivery Paradigms

For diseases requiring in vivo correction, AAVs remain one of the most widely used delivery platforms due to their ability to achieve efficient and tissue‐specific gene transfer. AAV‐mediated delivery has been widely explored for base editing applications targeting tissues such as the liver, muscle, retina and the central nervous system. However, the therapeutic use of AAVs is strongly shaped by tissue‐specific delivery demands and by the restricted cargo capacity of the vector, which is approximately 4.7 kb, whereas many base editors exceed 5–6 kb. As a result, AAV‐based base editing strategies generally fall into two categories: single‐AAV systems enabled by compact editor architectures, and dual‐AAV systems that rely on split‐intein or related reconstitution strategies to accommodate larger constructs. These complementary approaches have provided valuable platforms for evaluating base editing across diverse disease models.

Single‐AAV delivery strategies are enabled by the use of compact Cas variants, optimized base editor architectures or favourable construct design that allows the entire editing system to be packaged within a single vector. This approach simplifies vector design, reduces manufacturing complexity and facilitates more efficient in vivo delivery.

This strategy is exemplified in lysosomal storage diseases (LSDs), where early and efficient systemic delivery is critical for therapeutic efficacy. LSDs are inherited metabolic disorders marked by the buildup of undigested substrates inside lysosomes, caused by deficiencies in particular enzymes, resulting in cellular dysfunction and damage in multiple organs. A preclinical study demonstrated the potential of in utero adenine base editing to correct genetic mutations responsible for LSDs. In particular, the researchers focused on correcting the G to A (W392X) mutation in the *Idua* gene of a mouse model for Mucopolysaccharidosis type I (MPS‐IH), commonly known as Hurler syndrome [126]. This mutation causes a deficiency of α‐L‐iduronidase, leading to the buildup of glycosaminoglycans (GAGs) in multiple organs and resulting in severe systemic disease. Utilizing AAV9 vectors, the team delivered an ABE to the fetal mice at embryonic day 15.5. The ABE effectively corrected the W392X mutation in hepatocytes and cardiomyocytes, while a lower level of editing was detected in the brain. This correction correlated with marked improvements in survival rates and alleviation of metabolic, musculoskeletal and cardiac defects. Importantly, prenatal treatment reduced GAG buildup and improved cardiac function, highlighting the therapeutic promise of early genetic correction. This study demonstrates the potential of in utero base editing as a viable approach for treating genetic diseases prior to the appearance of clinical symptoms. Although this proof‐of‐concept study provides encouraging insights, additional research is needed to evaluate the safety, effectiveness and long‐term effects of these interventions in humans.

Similarly, single‐AAV delivery has been successfully applied in neuromuscular disorders such as Duchenne muscular dystrophy (DMD). DMD is a serious and advancing genetic condition linked to the X chromosome, resulting from mutations in the *DMD* gene responsible for producing dystrophin, a critical protein for muscle function. Mutations such as deletions, duplications, and point mutations cause the loss or malfunction of dystrophin, which leads to fragile muscle fibres, persistent inflammation fibrosis, and progressive muscle degeneration. Approximately 15%–20% of DMD occurrences are due to nonsense point mutations that introduce premature termination codons (PTCs), halting dystrophin translation [127]. ABE technology is well‐suited to correct many of these nonsense mutations (e.g., TAA>CAA, TAG>CAG, TGA>CGA) [128].

Several studies have explored ABE‐mediated correction of DMD nonsense mutations. An ABE fused to the SpG Cas9 variant (SpG‐ABE) which recognizes NGN PAMs was utilized to expand targeting possibilities [129]. Specific nonsense mutations (c.4174C>T creating a TAA stop codon; c.2977C>T creating a TGA stop codon) were targeted in DMD patient‐derived iPSC‐cardiomyocytes and in humanized mouse models carrying these mutations. AAV vectors were used for delivery, either via local intramuscular injection or systemic administration in neonatal mice. Efficient A•T to G•C editing was achieved in vitro (~58% in iPSCs) and in vivo (e.g., ~13% genomic editing in muscle after local delivery, ~37% in heart after systemic delivery). This correction led to substantial recovery of dystrophin expression in cardiomyocytes in vitro and in heart and skeletal muscle fibres in vivo (e.g., > 90% positive fibres in the heart after systemic delivery). Importantly, systemic ABE treatment in neonatal mice resulted in significant improvements in motor performance.

The nonsense mutation (CAA>TAA) in exon 53 of the commonly used mdx4cv mouse model was targeted for correction in a separate research using an improved ABE coupled to an NG‐PAM recognizing Cas9 (iABE‐NG) [127]. Systemic delivery via AAV9 in adult mice led to high levels of T•A to C•G editing at the target site, particularly in the heart, where editing reached ~85% at the mRNA level after 10 months. Consequently, dystrophin protein expression was restored to near‐complete levels in the heart, accompanied by significant enhancements in muscle contractile function.

Modification of splice donor sequences using base editing to induce exon skipping was demonstrated as an approach to recover the *DMD* gene reading frame in cases of exon deletions [130]. The researchers employed ABEs at key positions within splice donor sites, leading to the skipping of exon 50 or 45, depending on the mutation. This targeted editing successfully restored an in‐frame dystrophin transcript in patient‐derived iPSC‐cardiomyocytes and in a ΔEx51 mouse model. Using AAV9 vectors for in vivo delivery, the team achieved dystrophin restoration and phenotypic rescue in muscle tissue. The study demonstrated that base editing can precisely reprogram splicing patterns to correct frame‐disrupting mutations in *DMD*.

Systemic single‐AAV delivery has also demonstrated strong therapeutic potential in multisystem disorders such as Hutchinson–Gilford progeria syndrome (HGPS). HGPS is a rare and severe genetic disorder characterized by premature aging, mainly resulting from a single C•G to T•A point mutation (c.1824C>T, G608G) in the *LMNA* gene encoding nuclear lamin A. The mutation in *LMNA* exon 11 creates an abnormal splice site, leading to the production of a toxic truncated protein called progerin. Progerin accumulates in cells and drives disease pathology, with particularly severe effects on the vasculature. A landmark study demonstrated the clinical potential of in vivo base editing using a mouse model of HGPS that was homozygous for the human *LMNA* c.1824C>T mutation [109]. The researchers employed an ABE variant (ABE7.10max‐VRQR), designed to recognize an NGA PAM sequence adjacent to the mutation, to revert the pathogenic T•A base pair back to the wild‐type C•G. A single systemic administration of AAV9 encoding the ABE and a gRNA was delivered at postnatal day 14. Six months after treatment, substantial correction of the mutation was observed across multiple tissues, with editing efficiencies ranging from approximately 20%–60% and reaching ~23% in the critical aortic tissue. This genomic correction corresponded with reduced levels of aberrantly spliced *LMNA* mRNA and diminished progerin protein expression. Phenotypically, ABE‐treated mice showed striking improvements. The therapy preserved vascular smooth muscle cells (VSMCs) in the aorta and prevented adventitial fibrosis—two key pathological features of HGPS [131]. Most notably, a single ABE injection improved the median survival time of progeria mice from 215 days in untreated controls to 510 days—a 2.4‐fold increase approaching the age associated with the onset of old age in healthy mice. This study provided compelling evidence that in vivo base editing can directly repair the causative genetic defect in HGPS and deliver unprecedented therapeutic benefits in a preclinical model.

Beyond monogenic disorders, single AAV‐delivered base editing has also been explored in oncology models. In the context of hepatocellular carcinoma (HCC), a prevalent mutation in the *TERT* promoter, specifically the −124 C>T alteration, leads to increased telomerase activity and contributes to tumorigenesis. Researchers have developed a sgRNA‐guided *Campylobacter jejuni* Cas9 (CjCas9)‐fused ABE system capable of efficiently converting this mutation back to the wild‐type sequence in HCC cells [132]. This correction results in decreased TERT expression, reduced telomerase activity and inhibited tumour cell proliferation. In vivo experiments have shown the therapeutic promise of this base editing method. Delivery of the ABE system via AAV vectors to mouse models harbouring the *TERT* promoter mutation led to significant inhibition of liver tumour growth. The treated mice exhibited reduced tumour volumes and improved survival rates, emphasizing the efficacy of base editing in correcting oncogenic mutations and halting tumour progression. The therapeutic effect is driven by the targeted deamination of the mutated adenine to inosine leading to a permanent A‐to‐G base substitution. This targeted correction restores the normal transcription factor binding sites in the *TERT* promoter, thereby normalizing TERT expression levels. Likewise, local injection of AAVs delivering sgRNA‐guided CjABE into mouse models with the *TERT* promoter mutation led to significant suppression of brain tumour growth [133]. By leveraging the specificity and efficiency of ABEs, this approach provides a promising strategy for treating cancers driven by specific genetic mutations, opening new avenues for tailored and more efficient treatments.

In contrast to single‐AAV systems, many base editor constructs exceed the packaging capacity of AAV vectors, necessitating the use of dual‐AAV delivery strategies. In these approaches, the base editor is divided into two separate components, each packaged into an individual AAV vector, which reconstitute into a functional enzyme within the target cell through split‐intein or related protein reassembly mechanisms. While this strategy overcomes cargo size limitations and expands the range of targetable genomic loci, it introduces additional complexity, including the requirement for efficient co‐transduction of both vectors and potential variability in reconstitution efficiency.

This approach is exemplified in metabolic liver disorders such as phenylketonuria (PKU), where large base editor constructs require dual‐AAV delivery for effective in vivo correction.

PKU is a genetic condition resulting from mutations in the *phenylalanine hydroxylase* (*PAH*) gene, which causes increased phenylalanine levels and related neurological complications. A CBE approach was employed to correct a disease‐causing point mutation in the *Pah* gene associated with PKU [134]. Specifically, a split‐intein CBE system was utilized as a method to enable the delivery of the rather large base editor components through a dual AAV vector approach. When the AAV‐base editor system was delivered intravenously to adult Pah^enu2^ mice, a model of human PKU, the treatment successfully corrected the mutation, lowering blood phenylalanine levels to within the normal physiological range below 120 μmol/L. Significantly, mRNA correction rates reached as high as 63%, resulting in restored PAH enzyme function and the reversal of the light fur phenotype typical of PKU in these mice. These results highlight the practicality of employing AAV‐mediated base editing for in vivo correction of genetic mutations, presenting a promising therapeutic strategy for metabolic liver disorders such as PKU.

Similarly, dual‐AAV delivery has been applied in a gene therapy strategy used CBEs to silence the activity of the mutant *SOD1* gene by introducing a premature stop codon [135]. Because of the restricted loading capacity of AAV vectors, the researchers implemented an intein‐based split method to deliver the base editor in two parts using dual AAVs, which reassemble in vivo. Following intrathecal injection of these dual AAVs into G93A‐*SOD1* ALS mice, the split‐intein CBE successfully introduced a nonsense mutation in the target gene, causing a significant delay in disease progression. Treated mice showed prolonged survival, slower muscle atrophy, better neuromuscular function, reduced muscle denervation, and up to 40% fewer toxic SOD1 protein aggregates compared to untreated controls. This investigation emphasizes the capabilities of base editing specifically, split‐intein‐mediated delivery of CBEs as a viable therapeutic strategy for ALS and possibly other genetic diseases, overcoming vector size limitations while achieving precise, permanent gene silencing.

Dual‐AAV systems have also been successfully utilized in inherited retinal diseases such as Leber congenital amaurosis (LCA), where localized delivery enables efficient reconstitution of split base editors. LCA is a serious inherited retinal disorder caused by mutations in genes such as *RPE65*, resulting in early‐onset vision impairment. Two pivotal studies have examined the implementation of ABEs to correct mutations in the *Rpe65* gene, a typical reason for LCA, using the rd12 mouse model. While both studies employed ABEs delivered via AAV vectors, they employed distinct methodologies and targeted different aspects of visual function restoration, leading to varying outcomes.

The initial research centered on correcting the p.R44X nonsense mutation within the *Rpe65* gene [136]. In vivo base editing was performed using dual AAV9 vectors carrying split ABEs linked by a trans‐splicing intein. Subretinal delivery of these vectors into rd12 mice induced an A‐to‐G conversion in RPE cells, leading to the restoration of wild‐type *RPE65* mRNA and protein. This correction led to the recovery of light‐induced electrical responses in the retina, as measured by electroretinography (ERG), with sustained functional improvements observed for at least 3 months post‐treatment.

The objective of the second study was to protect cone photoreceptors, cells essential for colour perception and sharp vision [137]. The study involved laboratory screening (in vitro) to pinpoint the most effective ABE/sgRNA combinations for fixing the *Rpe65* mutation. Delivery of the optimized ABE and sgRNA through split AAV2 vectors via subretinal injection corrected up to 40% of *Rpe65* transcripts, restored cone‐mediated vision and preserved cone cells in rd12 mice. Additional analysis with single‐cell RNA sequencing showed increased expression of genes linked to cone phototransduction and survival pathways in the treated mice. These findings suggest that base editing not only corrects genetic mutations but also promotes the expression of genes critical for cone function and longevity. The study showed that base editing therapy can provide long‐term restoration of cone photoreceptor function and survival.

Together, these studies demonstrate that dual‐AAV strategies provide a practical solution to AAV cargo limitations, enabling the use of full‐length or less compact base editors across diverse tissues. However, compared to single‐AAV systems, they introduce additional delivery complexity and depend on efficient co‐transduction, which may affect overall editing efficiency and translational scalability.

#### 6.2.4 Transient Potency: LNPs and Liver Targeting

LNPs have emerged as one of the most promising delivery platforms for in vivo base editing, particularly for liver‐targeted therapies. LNPs can efficiently encapsulate mRNA encoding base editors together with gRNAs and deliver them to hepatocytes following systemic administration. This transient delivery approach avoids long‐term nuclease expression and reduces the risk of integration‐related complications. The primary trade‐off in this paradigm involves delivery safety versus durable efficacy.

One of the most advanced applications of LNP‐mediated base editing targets is the *PCSK9* gene (Proprotein Convertase Subtilisin/Kexin type 9), a well‐validated regulator of LDL‐C levels. The primary goal is to achieve durable LDL‐C reduction through permanent gene inactivation in the liver, offering a potential single‐treatment alternative to chronic therapies like antibodies or siRNA. ABEs are particularly suited for this application. These editors are often delivered via LNPs that efficiently target hepatocytes, aiming to introduce a specific base change at a critical *PCSK9* splice site, thereby disrupting gene function and preventing the production of the PCSK9 protein.

Pivotal preclinical validation came from studies in non‐human primates (NHPs), primarily cynomolgus monkeys [138]. Researchers employed ABEs to precisely edit the *PCSK9* gene in the livers of mice and cynomolgus macaques. Delivered via LNPs, the ABE system achieved up to 67% editing efficiency in mice and 34% in macaques. This resulted in a 95% decrease in plasma PCSK9 levels and a 58% reduction in LDL cholesterol in mice, and a 32% reduction in PCSK9 and 14% in LDL cholesterol in macaques. Importantly, the ABE mRNA was rapidly cleared from the system, and no unintended mutations were observed. However, an antibody‐mediated immune response against the ABE was observed upon re‐dosing in macaques. These results highlight the promise of ABE‐mediated gene editing as a therapeutic approach for treating monogenic liver diseases, such as familial hypercholesterolemia.

The use of in vivo CRISPR base editing aiming at the *PCSK9* gene was also explored in primates as a strategy to reduce cholesterol levels [139]. Following a single intravenous infusion of LNPs carrying the base editor, an almost complete suppression of *PCSK9* expression was achieved in the liver. This resulted in roughly a 90% decrease in circulating PCSK9 protein and about a 60% reduction in LDL cholesterol, with these effects remaining stable for at least 8 months. The findings support a promising one‐time intervention for lowering LDL cholesterol and managing atherosclerotic cardiovascular disease (ASCVD). Furthermore, this study offers strong proof‐of‐concept for using CRISPR base editors to perform precise single‐nucleotide modifications in disease‐related genes within the liver, and potentially in other tissues.

ASCVD encompasses conditions resulting from plaque accumulation in arterial walls, which narrows or blocks arteries and raises the risk of heart attack, peripheral artery disease and stroke [140]. Base editing, a technology advanced by companies such as Verve Therapeutics, presents a promising therapeutic strategy for ASCVD due to its capacity for precise and permanent correction of genetic variants associated with increased cardiovascular risk. The initial programs focus on three key genetic targets associated with elevated cholesterol and ASCVD: the *PCSK9* gene to reduce LDL cholesterol, the *ANGPTL3* (angiopoietin‐like protein 3) gene to lower triglycerides and remnant cholesterol and the *LPA* gene to decrease lipoprotein(a) levels [141, 142, 143].

The key strategy involves using insights from human genetics to mimic the protective effects of natural gene variants, like *PCSK9* loss‐of‐function mutations known to cause lifelong low LDL‐C levels and lower ASCVD risk [144]. VERVE‐101, the first clinical candidate, employs an ABE delivered via LNPs to inactivate the *PCSK9* gene in the liver by introducing a targeted A•T to G•C base pair substitution [145]. The therapeutic goal was to achieve permanent reductions in LDL‐cholesterol for patients suffering from Heterozygous Familial Hypercholesterolemia (HeFH) and established ASCVD. VERVE‐101 consists of two components: a gRNA that targets the splice donor site between exon 1 and intron 1 of the *PCSK9* gene to introduce a premature stop codon, thereby halting the production of functional PCSK9 protein, and an mRNA encoding the ABE8.8 m enzyme [146]. This base editor comprises a modified SpCas9 protein fused to an engineered TadA adenosine deaminase domain.

The Phase 1b heart‐1 trial (NCT05398029), launched in 2022, provided the first proof‐of‐concept for in vivo base editing in humans and showed encouraging early outcomes in reducing LDL‐C levels [147]. Interim data from the human trial, released in late 2023, showed dose‐dependent decreases in blood PCSK9 protein and LDL‐C levels [148]. Notably, the single participant in the highest dose group (0.6 mg/kg) exhibited a 55% reduction in LDL‐C that was sustained for at least 18 months. However, safety issues emerged in this high‐risk patient group during the trial. Findings encompassed temporary elevations in liver enzymes, along with two serious cardiovascular events: a fatal cardiac arrest (concluded unrelated to treatment due to the patient's severe pre‐existing heart condition) and a heart attack with a possible connection to the treatment. Additionally, later laboratory tests identified low platelet counts (thrombocytopenia) and elevated liver function tests (LFTs), which were considered likely side effects of the particular LNP delivery system used in VERVE‐101 [149]. Due to these findings, enrollment in the heart‐1 trial was halted.

The occurrence of serious cardiovascular events illustrates the inherent complexity of conducting first‐in‐human gene editing trials in patients with severe, advanced ASCVD. In this high‐risk population, the frequent incidence of events such as myocardial infarction and cardiac arrest complicates the assessment of whether these outcomes are linked to the investigational therapy. Although the myocardial infarction soon after dosing was tentatively linked to the treatment, the fatal cardiac arrest occurring weeks later emphasizes the challenge of separating therapy‐induced events from the expected progression of severe cardiovascular disease. Investigations, which included nonclinical studies, determined that the observed laboratory abnormalities specifically elevated liver enzymes and thrombocytopenia were likely driven mainly by the LNP delivery system used in VERVE‐101, rather than the base editing components themselves.

Although regulatory filings for VERVE‐101 are still ongoing, greater emphasis is now being placed on VERVE‐102. This drug candidate employs the same *PCSK9*‐targeting base editor and gRNA as VERVE‐101 but is delivered through a different LNP system [150]. The ionizable lipid included in this alternative LNP formulation has a history of previous use and good tolerability in other clinical trials. Unlike LNPs relying solely on ApoE for uptake via the LDLR, the VERVE‐102 LNP incorporates the targeting ligand N‐acetylgalactosamine (GalNAc) to achieve more specific and efficient delivery to the liver [151]. This improved targeting could potentially allow for lower effective doses or enhance the therapeutic index by minimizing exposure to non‐target cells. VERVE‐102 is set to be evaluated in patients with HeFH and ASCVD in the Phase 1b Heart‐2 trial (NCT06164730).

Additionally, VERVE‐201, an investigational therapy which also uses LNP‐delivered base editing but targets the distinct *ANGPTL3* gene, is being advanced through preclinical development [152]. The *ANGPTL3* gene plays a central role in regulating cholesterol and triglyceride levels, contributing to the risk of ASCVD via a mechanism distinct from the PCSK9 pathway. ANGPTL3, a liver‐derived protein, inhibits two essential enzymes involved in lipid metabolism: lipoprotein lipase (LPL) and endothelial lipase (EL). By inhibiting LPL and EL, ANGPTL3 increases levels of triglycerides (TG), cholesterol‐rich remnant lipoproteins, as well as LDL‐C and HDL‐C. Suppressing ANGPTL3 protein production in the liver has been shown to lower both LDL‐C and TG concentrations [153]. The fact that individuals naturally lacking a working *ANGPTL3* gene have much lower LDL‐C and triglycerides without obvious health problems indicates that targeting this gene for therapy could be both effective and safe [154]. VERVE‐201 employs the same GalNAc‐LNP delivery system utilized for VERVE‐102, which incorporates the clinically validated ionizable lipid and the GalNAc targeting ligand for efficient and specific hepatocyte delivery. Clinical trials for VERVE‐201 are anticipated to begin soon. Success in these programs could mark a new era in cardiovascular medicine, moving from chronic disease management to long‐term disease modification through precision genetic medicine.

Similarly, GSD1a is a rare inherited metabolic disorder with autosomal recessive inheritance, caused by mutations in the *G6PC* gene that result in deficient activity of the glucose‐6‐phosphatase (G6Pase) enzyme [155]. This enzyme deficiency disrupts the final stages of glycogenolysis and gluconeogenesis, impairing the body's ability to regulate glucose levels. GSD1a typically presents in early infancy with severe fasting hypoglycemia, lactic acidosis, hyperlipidemia, hyperuricemia and notable hepatomegaly and nephromegaly due to glycogen and fat buildup. Long‐term complications can include hepatic adenomas with a risk of malignant transformation, progressive renal disease, osteoporosis and gout. The R83C mutation in the *G6PC* gene, common in Caucasian populations including those of Ashkenazi Jewish descent, causes a complete loss of G6Pase enzyme activity, contributing to the severe clinical phenotype of GSD1a [115]. The GSD1a‐causing R83C mutation arises from a G>A transition on the coding strand of the *G6PC* gene. Consequently, the complementary non‐coding strand carries a C>T change at the corresponding position. To restore the wild‐type *G6PC* sequence, this aberrant T on the non‐coding strand must be converted back to a C. This requires changing the A•T base pair at the mutation site back to the original G•C base pair.

ABE technology is well‐suited for correcting the R83C mutation in GSD1a. BEAM‐301, developed by Beam Therapeutics and delivered via LNPs, uses ABE to revert the A•T base pair to the wild‐type G•C pair. This precise correction restores G6Pase enzyme activity and addresses the metabolic imbalances associated with GSD1a [100]. A Phase 1/2 clinical trial evaluating the dose, safety, and efficacy of BEAM‐301 (NCT06735755) is currently underway. This follows the FDA's clearance of the Investigational New Drug (IND) application for the therapy in the second quarter of 2024. BEAM‐301 holds significant potential as an advancement in treating GSD1a, shifting from palliative dietary management to a possible one‐time genetic cure. If successful, this in vivo base editing therapy could fundamentally alter the disease's progression, relieving patients from lifelong dietary restrictions and reducing the risk of severe hypoglycemia and long‐term organ complications.

Another liver‐targeted base editing therapy currently under development addresses alpha‐1 antitrypsin deficiency (AATD). AATD is a genetic metabolic disorder characterized by reduced circulating levels of alpha‐1 antitrypsin (AAT) protein, leading to an increased risk of serious lung and liver diseases [116]. AAT is a serine protease inhibitor (serpin) predominantly produced and secreted by liver cells. Its main role is to inhibit neutrophil elastase in the lungs, protecting the delicate alveolar tissue from proteolytic damage, particularly during inflammatory responses. Mutations in the *SERPINA1* gene on chromosome 14q32 cause AATD, with the *PiZ* allele identified as the most clinically significant variant [117]. The *PiZ* allele results from a single point mutation (c.1096G>A) that substitutes glutamic acid with lysine at codon 342, causing the AAT protein (Z‐AAT) to misfold and accumulate in the liver, leading to hepatocyte stress, inflammation and an increased risk of cirrhosis and liver cancer. This misfolding also impairs AAT secretion, drastically reducing circulating AAT levels, which leaves the lungs vulnerable to neutrophil elastase, resulting in progressive alveolar damage, early‐onset emphysema and potentially bronchiectasis.

The *PiZ* mutation in AATD is a G>A transition. ABE technology is ideally suited for correcting this specific type of mutation [118]. Using in vivo base editing, BEAM‐302 is an investigational therapy from Beam Therapeutics designed to target the genetic origin of AATD. The therapeutic strategy centers on correcting the specific *PiZ* point mutation (G>A) in the *SERPINA1* gene directly within the patient's liver cells [100]. The base editing components, mRNA encoding the ABE and gRNA targeting the *PiZ* mutation, are delivered via LNPs. After intravenous administration, the LNPs are designed to preferentially accumulate in the liver. Hepatocyte uptake is facilitated by ApoE adsorbed on the LNP surface, which interacts with LDLR on hepatocytes. Once internalized through endocytosis, the LNPs release their mRNA and gRNA cargo into the cytoplasm, where the mRNA is translated into the ABE protein. The ABE then binds to the gRNA to form a complex capable of editing the target DNA. Correction of the *PiZ* mutation reduces toxic Z‐AAT accumulation in hepatocytes and restores normal M‐AAT production. By targeting the gene's native locus, this approach enables physiologically regulated expression, boosts protective AAT levels and may provide a durable, one‐time treatment.

Building on promising preclinical results, a Phase 1/2 open‐label, multicenter clinical trial (NCT06389877) was initiated to assess BEAM‐302 in adults with AATD. The study comprises dose‐escalation (Phase 1) and dose‐expansion (Phase 2) phases, aimed at assessing the safety, tolerability, pharmacokinetics and therapeutic efficacy of single ascending doses, while determining the optimal biological dose (OBD) for further development. Dosing in the Phase 1/2 trial commenced in mid‐2024 [119]. Initial data from the first cohorts (released March 2025) showed dose‐dependent increases in functional AAT (reaching therapeutic levels), significant reductions in the mutant protein and a favourable safety profile (mild/moderate transient events) [156]. Ongoing studies focusing on dose Optimisation, long‐term outcomes, and patients with liver involvement will be key to defining its full clinical potential. If successful, BEAM‐302 could shift AATD management from ongoing symptomatic care to a durable, gene therapy solution.

#### 6.2.5 Rapid Response: The Personalized Paradigm

The ultimate synthesis of base editing's modularity and delivery efficiency is seen in ultra‐rare, personalized cases. For a neonate with carbamoyl‐phosphate synthetase 1 (CPS1) deficiency, a personalized in vivo therapy named kayjayguran abengcemeran (k‐abe) was designed to correct the patient's paternal CPS1 variant (Q335X) [157]. The therapy, consisting of mRNA encoding the ABE and a custom gRNA delivered via LNPs, progressed from genetic diagnosis through preclinical validation in patient‐derived cells and animal models (including mouse efficacy and nonhuman primate toxicology studies) to IND application approval and clinical administration within approximately 7 months.

The patient received two IV infusions of k‐abe at approximately 7 and 8 months of age, starting with a 0.1 mg/kg dose followed by 0.3 mg/kg. Following treatment, the patient demonstrated notable clinical improvements, including the ability to tolerate increased dietary protein and a 50% reduction in the required dose of nitrogen‐scavenger medication. A significant outcome was the infant's ability to endure subsequent viral illnesses without developing hyperammonemic crises, which had previously been a substantial risk. While transient elevations in liver transaminases were observed, no serious adverse events occurred. Although long‐term safety and efficacy require further monitoring, this case establishes a new paradigm for the rapid development and deployment of customized gene‐editing therapies for individuals with rare or unique genetic diseases.

This study's primary significance lies in demonstrating the feasibility of developing and deploying a personalized, in vivo gene‐editing therapy with unprecedented speed within just 6 months of a patient's birth. This work validates a crucial proof‐of‐concept workflow, demonstrating a platform approach that enables the rapid creation of customized treatments for ultrarare genetic diseases by modifying only specific components, such as the gRNA. This success paves the way for a future where rapid deployment of patient‐specific gene‐editing therapies could become a routine strategy for managing a wide array of genetic disorders [158].

Taken together, these delivery platforms highlight fundamental trade‐offs that shape the clinical translation of base editing technologies. Preclinical approaches such as hydrodynamic tail‐vein injection provide valuable proof‐of‐concept for efficient in vivo editing but lack direct clinical applicability. Ex vivo strategies offer the highest level of control over editing outcomes, enabling precise quality assessment and selection of modified cells prior to reinfusion. However, they are limited to clinically accessible cell types and require complex manufacturing workflows. In contrast, in vivo delivery approaches expand therapeutic reach. AAV‐mediated systems enable efficient gene transfer across diverse tissues, including muscle, retina and the central nervous system, and have already demonstrated clinical feasibility through multiple approved gene therapies. However, they are constrained by limited cargo capacity and prolonged transgene expression, often necessitating dual‐vector strategies. LNP‐based delivery, particularly for liver‐targeted applications, provides a clinically scalable and non‐viral alternative, enabling transient expression of base editing components and broader patient applicability. Notably, LNP‐mediated base editing has already entered human clinical trials targeting genes such as *PCSK9* and has also enabled rapid, personalized therapeutic approaches, as exemplified in CPS1 deficiency. However, this approach offers reduced control over cell‐specific editing outcomes and may introduce delivery‐associated toxicities. Collectively, these platforms emphasize the importance of aligning delivery modality with disease biology, tissue accessibility and therapeutic objectives to maximize the clinical impact of base editing interventions.

## 7 Conclusion

### 7.1 The Transformative Impact of Base Editing

In summary, CRISPR‐based base editing has rapidly emerged as a transformative technology in genome engineering. Its capacity for direct and precise single DNA base conversion represents a significant leap forward, bypassing the need for DSBs and inefficient HDR processes common to conventional CRISPR‐Cas9 editing [159]. With the advancement of editors targeting both cytosine (C•G → T•A) and adenine (A•T → G•C) base conversions, along with emerging tools enabling a broader range of modifications, base editing holds great promise for correcting a substantial proportion of point mutations responsible for human genetic diseases [160]. Technology's rapid progression from concept to clinical trials within a decade highlights its immense potential and ongoing scientific appeal [161].

### 7.2 Advantages and the Path Forward

Base editing has gained rapid acceptance due to its ability to introduce precise nucleotide changes with improved predictability compared with nuclease‐based genome editing approaches (Table 4) [162]. However, realizing the full therapeutic potential of base editing requires overcoming key challenges [20]. Achieving the critical goal of near‐perfect specificity requires overcoming key challenges through robust strategies, namely minimizing off‐target edits (on DNA and RNA, Cas‐dependent or independent) and preventing unintended ‘bystander’ mutations close to the target [73]. While significant progress has been made in engineering higher‐fidelity editors, continuous rigorous assessment is vital. Arguably, the most substantial hurdle for in vivo therapies is developing safe and efficient delivery systems that can target tissues beyond the liver [163]. Additionally, managing potential immune responses to the editor components or the delivery vehicle is essential for ensuring long‐term safety and efficacy [164, 165].

**TABLE 4:** Comparative analysis of major genome editing platforms and their clinical maturity.

Editing platform	PAM requirement	Editing window	Bystander editing risk	Off‐target effects (DNA/RNA)	Editing scope	Delivery compatibility	Clinical status
Cytosine Base Editors (CBE)	NGG (SpCas9); expanded with SpG/SpRY	~4–8 nt (position‐dependent)	High (multiple cytosines editable)	DNA off‐target (Cas‐dependent + deaminase‐dependent); RNA off‐target (APOBEC)	C•G → T•A (transition)	LNP, AAV (dual), RNP, eVLPs	Advanced (Multiple Phase 1/2 trials ongoing)
Adenine Base Editors (ABE)	NGG; expanded PAM variants available	~4–7 nt	Moderate (adenine‐specific)	Lower DNA off‐target; RNA off‐target (reduced in evolved TadA‐8e variants)	A•T → G•C (transition)	LNP, AAV (dual), RNP, eVLPs	Advanced (Phase 1/2 trials ongoing)
Dual Base Editors	NGG; PAM‐flexible variants available	Overlapping or expanded windows	Higher (multiple editable bases and types)	Combined DNA/RNA off‐target risks from both deaminases	Simultaneous C → T and A → G editing (multiplexed transitions)	LNP, AAV (dual), RNP	Preclinical/early translational
Glycosylase‐Based Base Editors	NGG; PAM‐flexible variants emerging	Context‐dependent	Variable (dependent on repair outcomes)	Primarily DNA repair‐mediated; higher indel risk compared to ABE/CBE	Transversions (e.g., C → G, G → T, T → G)	LNP, RNP (delivery Optimisation ongoing)	Early‐stage (preclinical)
Prime Editors (PE)	NGG (expanded variants available)	No fixed window (pegRNA‐defined)	Minimal (single defined edit)	Low DNA off‐target; minimal RNA off‐target	All 12 substitutions, small insertions/deletions	LNP, AAV (dual), plasmid, RNP	Early clinical (first Phase 1/2 trial active)
CRISPR‐Cas9	NGG (or variant‐dependent)	Not applicable (DSB‐based)	Not applicable (Non‐specific indels)	High risk of indels, large deletions, chromosomal rearrangements	Indels, gene knockout, HDR‐based precise edits	Broad (viral, non‐viral)	Clinically established (Approved therapies, e.g., Casgevy; many Phase 3 trials)

*Note:* This table provides a comprehensive synthetic framework for evaluating the performance characteristics and clinical readiness of primary genome editing technologies. Major editor classes, including Cytosine Base Editors (CBEs), Adenine Base Editors (ABEs), Dual Base Editors and Glycosylase‐based variants, are cross‐evaluated against Prime Editors (PEs) and conventional CRISPR‐Cas9 nucleases. The comparison is structured around core performance trade‐offs, such as the flexibility of Protospacer Adjacent Motif (PAM) requirements, the precision of the editing window, and the liability of bystander editing. Additionally, the framework details DNA/RNA off‐target profiles, the specific scope of nucleotide modifications (e.g., transitions vs. transversions) and the current maturity of clinical validation as of March 2026. This synthesis is intended to guide the selection of appropriate editing platforms based on therapeutic modality and the specific genetic architecture of target mutations.

Abbreviations: ABE, Adenine Base Editor; APOBEC, Apolipoprotein B mRNA Editing enzyme Catalytic polypeptide‐like; Cas9, CRISPR‐Associated protein 9; CRISPR, Clustered Regularly Interspaced Short Palindromic Repeats; DSB, Double‐Strand Break; eVLP, engineered Virus‐Like Particle; HDR, Homology‐Directed Repair; Indels, Insertions and Deletions; LNP, Lipid Nanoparticle; nt, nucleotide; PAM, Protospacer Adjacent Motif; PE, Prime Editor; pegRNA, Prime Editing Guide RNA; RNP, Ribonucleoprotein; TadA, tRNA Adenine Deaminase.

### 7.3 Navigating the Ethical Landscape

Because base editing offers a remarkable power to precisely alter genetic information, its continued development demands careful ethical consideration [166]. Ensuring patient safety through rigorous assessment of all potential effects before clinical use is a core ethical responsibility. Furthermore, tackling questions of justice and how to provide equitable access to these potentially life‐changing, but probably expensive, therapies is essential [167]. A key ethical distinction lies between somatic (therapeutic) modifications, which affect only the treated individual and germline (heritable) modifications, which are passed on to future generations. This distinction is critical because germline changes carry unknown long‐term risks, raise concerns about consent for future generations and could be misused for human enhancement or eugenics, whereas somatic editing is generally considered ethically permissible if safety and efficacy are demonstrated [168].

Key ethical dilemmas include the risk of off‐target or unintended genetic changes, challenges in obtaining informed consent, particularly for vulnerable populations, and equitable access to potentially costly therapies. Societal implications are broad, encompassing public perception, cultural and religious perspectives, potential exacerbation of health disparities and the ethical debates surrounding human enhancement.

The ethical debate surrounding human genome editing was further intensified following the 2018 case of Chinese researcher He Jiankui, who reported the birth of the first gene‐edited babies after germline CRISPR‐Cas9 modification of the *CCR5* gene. This experiment, which aimed to confer resistance to HIV infection, was conducted without adequate scientific transparency or internationally accepted ethical oversight [169]. The announcement was widely condemned by the global scientific community and raised serious concerns regarding patient safety, the responsible conduct of research and the ethical legitimacy of heritable genome editing. Beyond the immediate scientific controversy, the incident underlined broader concerns about the premature clinical application of genome editing technologies and emphasized the urgent need for robust international governance frameworks and clearer regulatory oversight in human genome editing research [170].

In the wake of this controversy, global policy and scientific communities intensified efforts to establish governance frameworks for responsible genome editing research. International organizations and regulatory agencies have since emphasized the need for transparent oversight, ethical review and public engagement in the development and application of genome editing technologies. For instance, the World Health Organization (WHO) has established an Expert Advisory Committee on Developing Global Standards for Governance and Oversight of Human Genome Editing [171]. This committee has issued recommendations emphasizing the need for international collaboration, the establishment of human genome editing registries and the development of ethical principles to guide the use of these technologies. It has also highlighted the importance of education, engagement, and empowerment to ensure that all stakeholders are informed and involved in decision‐making processes.

Additionally, the National Institutes of Health (NIH) has emphasized the importance of equitable access to gene‐editing technologies, advocating for policies that ensure benefits reach all populations, particularly underserved communities, to prevent exacerbation of existing health disparities [172]. Furthermore, the Third International Summit on Human Genome Editing, held in 2023, emphasized that heritable human genome editing remains unacceptable at this time [173]. The summit's organizing committee called for ongoing dialogue and international collaboration to develop governance frameworks and ethical principles for the responsible use of heritable human genome editing. It also stressed the need for rigorous oversight and the establishment of reasonable standards for safety and efficacy before any clinical application.

Apart from regulatory issues, concerns about access and affordability are becoming increasingly central to ethical debates over gene‐editing therapies. Several recently approved gene therapies have treatment costs exceeding one million US dollars, raising concerns about global inequalities in access to advanced genetic medicine [174]. Addressing these challenges will likely require coordinated policy efforts, innovative pricing models and international collaboration to ensure equitable access to emerging genome‐editing therapies [175]. In addition, emerging personalized genome‐editing strategies, often described as individualized therapies, introduce new ethical and regulatory complexities. Approaches such as k‐ABE illustrate the potential of highly patient‐specific treatments designed to correct rare genetic variants affecting individual patients [139]. While such strategies may offer transformative benefits for otherwise untreatable diseases, they also raise unresolved questions regarding regulatory oversight, long‐term safety evaluation, cost sustainability and equitable access to individualized genome‐editing interventions [176]. Overall, addressing these considerations is essential to ensure that base editing is developed and applied safely, responsibly and in a manner that maximizes societal benefit while minimizing harm.

### 7.4 The Promising Future of Base Editing

The field of base editing is poised for continued rapid advancement. Future research is expected to prioritize next‐generation editors with enhanced precision, reduced off‐target activity, expanded targeting capabilities (such as broader PAM compatibility and reliable transversion editing) and potentially novel functionalities. Arguably, the most critical step towards wider therapeutic application beyond liver‐directed and ex vivo approaches is overcoming the in vivo delivery challenge [177]. Innovations in engineering LNPs for extrahepatic targeting, advancing AAV vector design (e.g., through capsid modifications or self‐limiting expression) and developing new non‐viral delivery platforms will be pivotal [178, 179]. Outcomes from ongoing clinical trials for diseases including SCD, T‐ALL, AATD, GSD1a and cardiovascular disease will provide essential insights into the long‐term safety and efficacy of base editing in humans, requiring rigorous monitoring for off‐target effects, immunogenicity and duration of the clinical benefit [100, 177, 180]. As technology matures alongside delivery methods, base editing holds immense potential to transition from a sophisticated research tool into a potentially curative therapy for a wide array of genetic disorders, fundamentally reshaping the future of medicine. Realizing this transformative potential, nevertheless, requires both sustained scientific progress and a firm dedication to safety and ethical principles.

## Author Contributions

**Ezgi Erbasan:** writing – original draft, validation. **Melike Aliciaslan:** writing – original draft, visualization, validation. **Fulya Erendor:** writing – original draft, visualization, validation. **Salih Sanlioglu:** writing – original draft, visualization, validation.

## Funding

The authors have nothing to report.

## Ethics Statement

The authors have nothing to report.

## Consent

The authors have nothing to report.

## Conflicts of Interest

The authors declare no conflicts of interest.

## Data Availability

The authors have nothing to report.
